# Supplementary figures and images for: Genome sequence analysis of Malayan pangolin (Manis javanica) forensic samples reveals the presence of Paraburkholderia fungorum sequences
Source: PeerJ. 2023 Oct 4;11:e16002. doi: 10.7717/peerj.16002 (PMC10559893; doi:10.7717/peerj.16002)

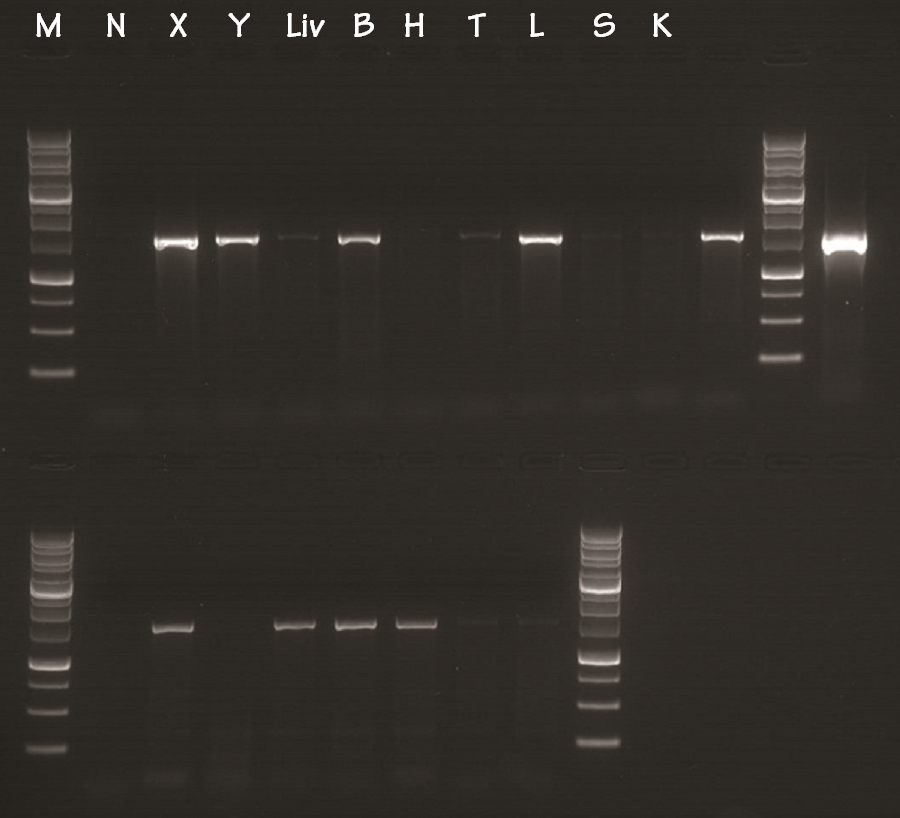

Supplement: Supplemental Information 4 [file peerj-11-16002-s004.zip › Figure 1/Target 16S/Target 16S labelled.png]

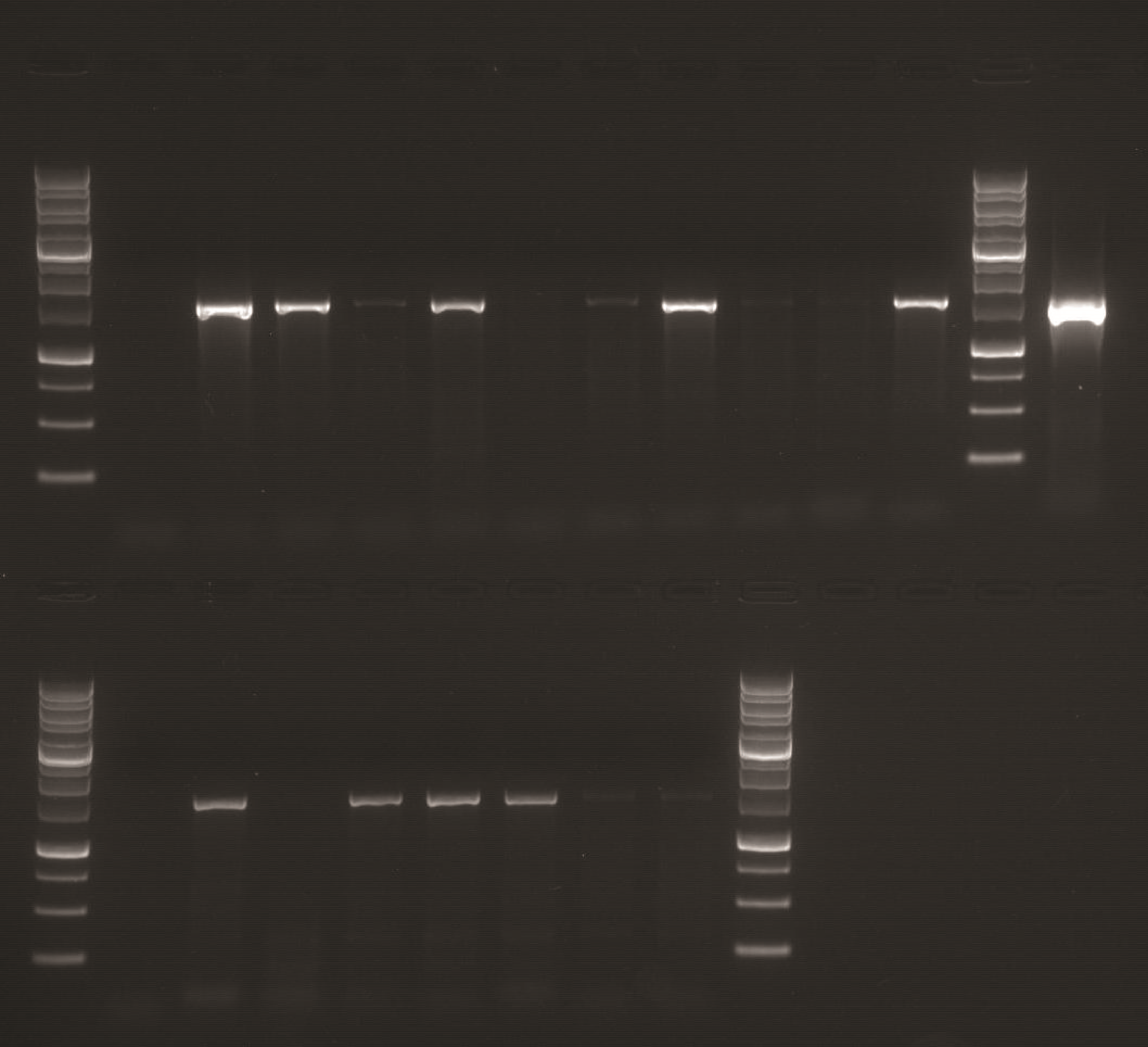

Supplement: Supplemental Information 4 [file peerj-11-16002-s004.zip › Figure 1/Target 16S/Target 16S.png]

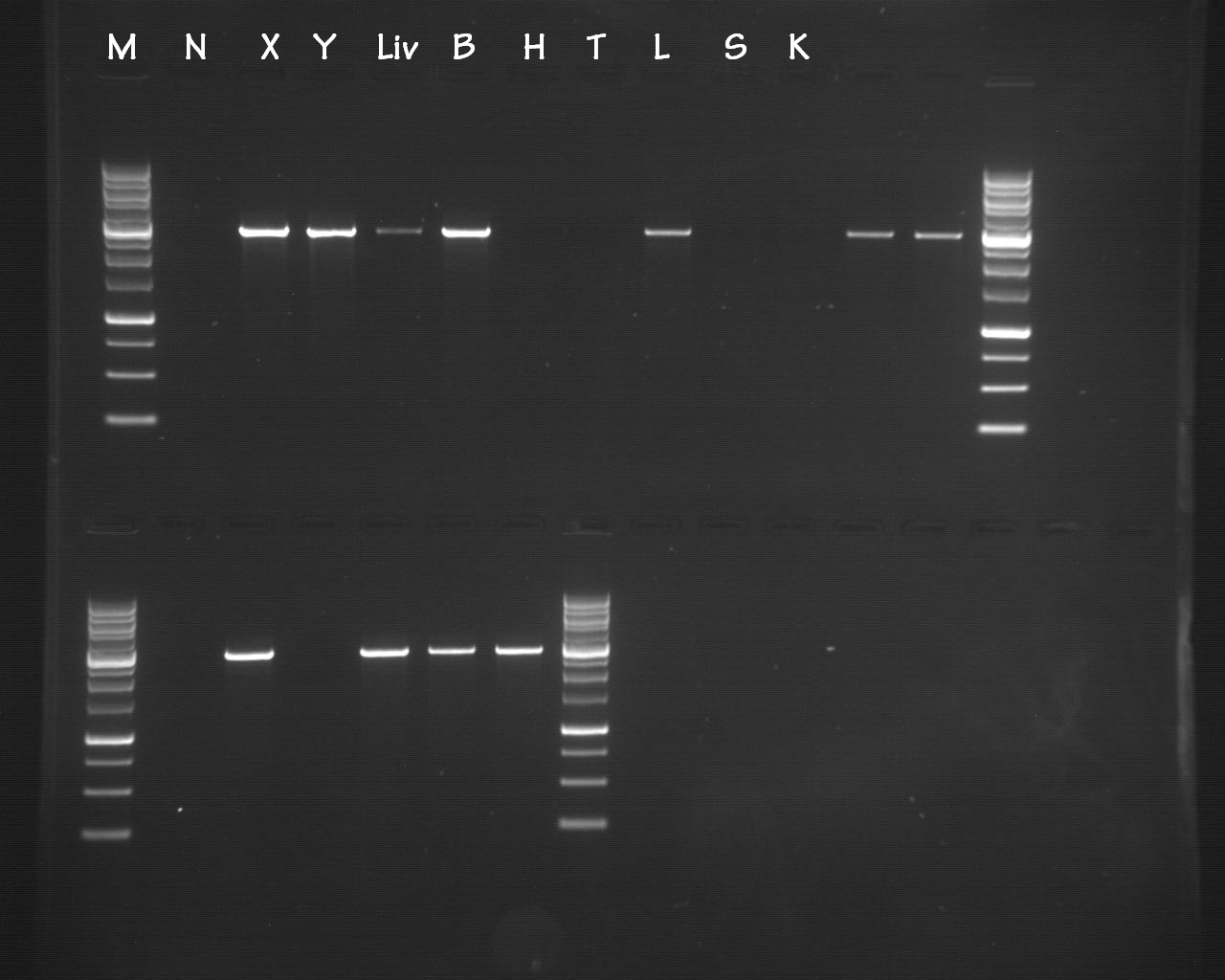

Supplement: Supplemental Information 4 [file peerj-11-16002-s004.zip › Figure 1/Target A/Target A labelled.png]

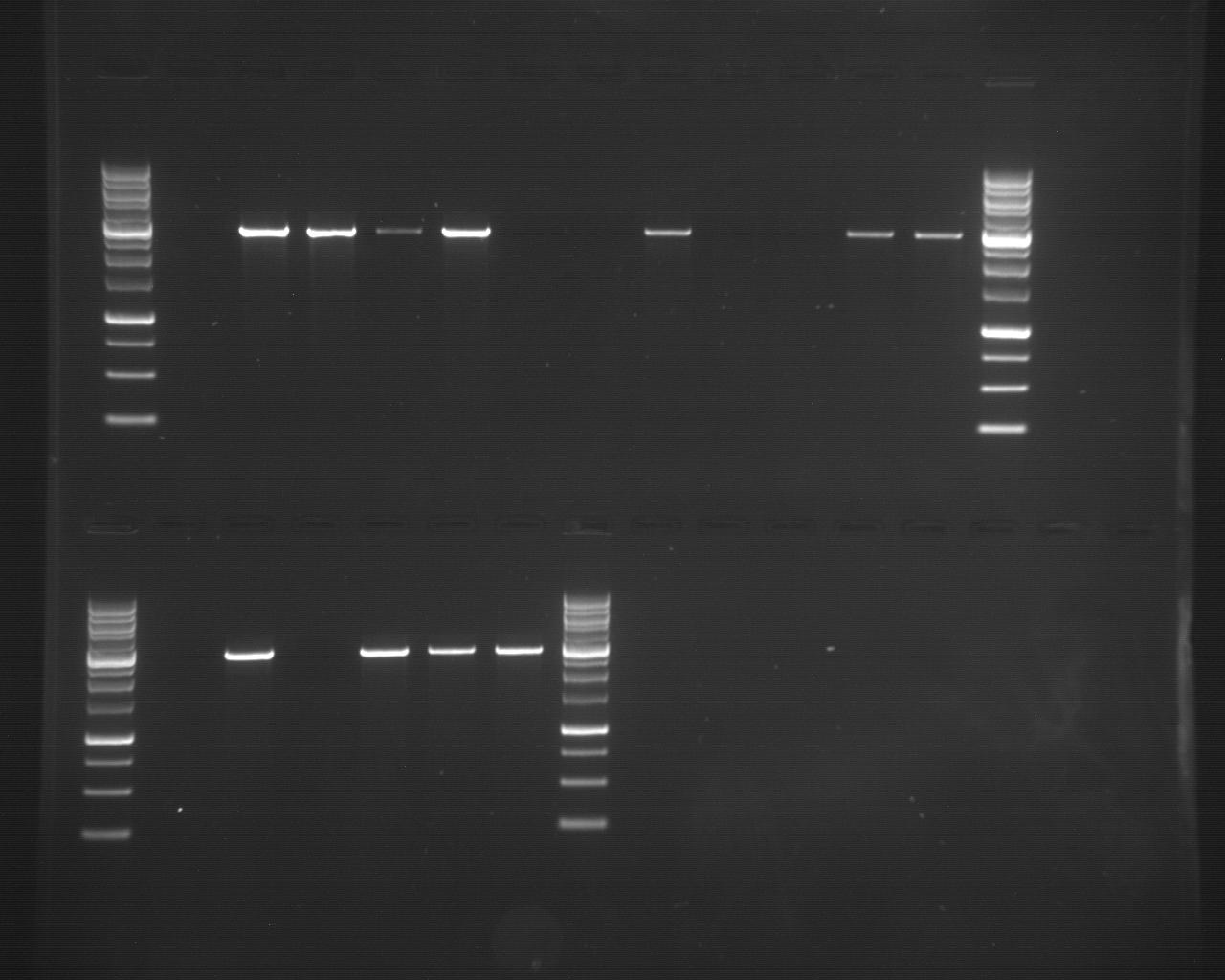

Supplement: Supplemental Information 4 [file peerj-11-16002-s004.zip › Figure 1/Target A/Target A.png]

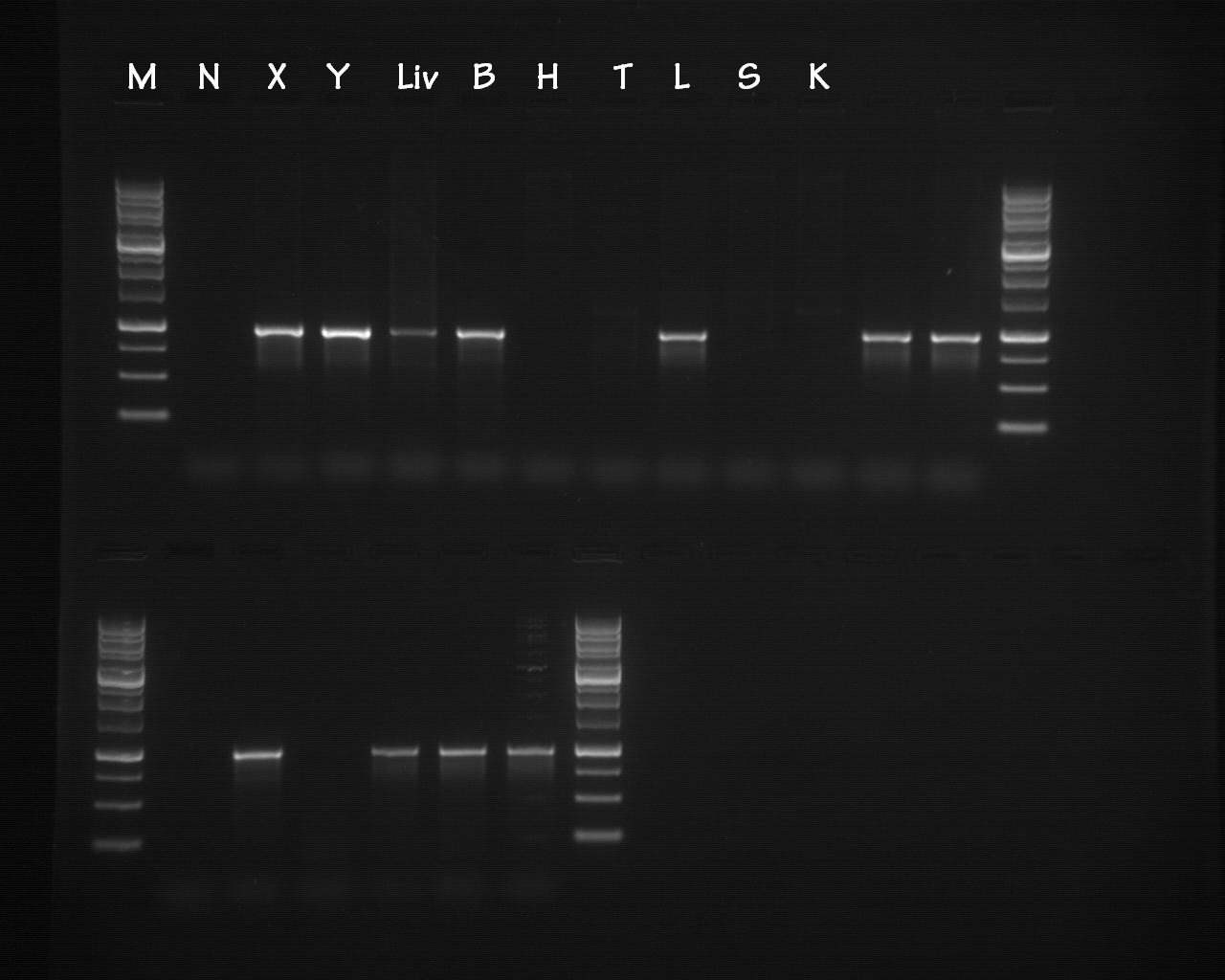

Supplement: Supplemental Information 4 [file peerj-11-16002-s004.zip › Figure 1/Target B/Target B labelled.png]

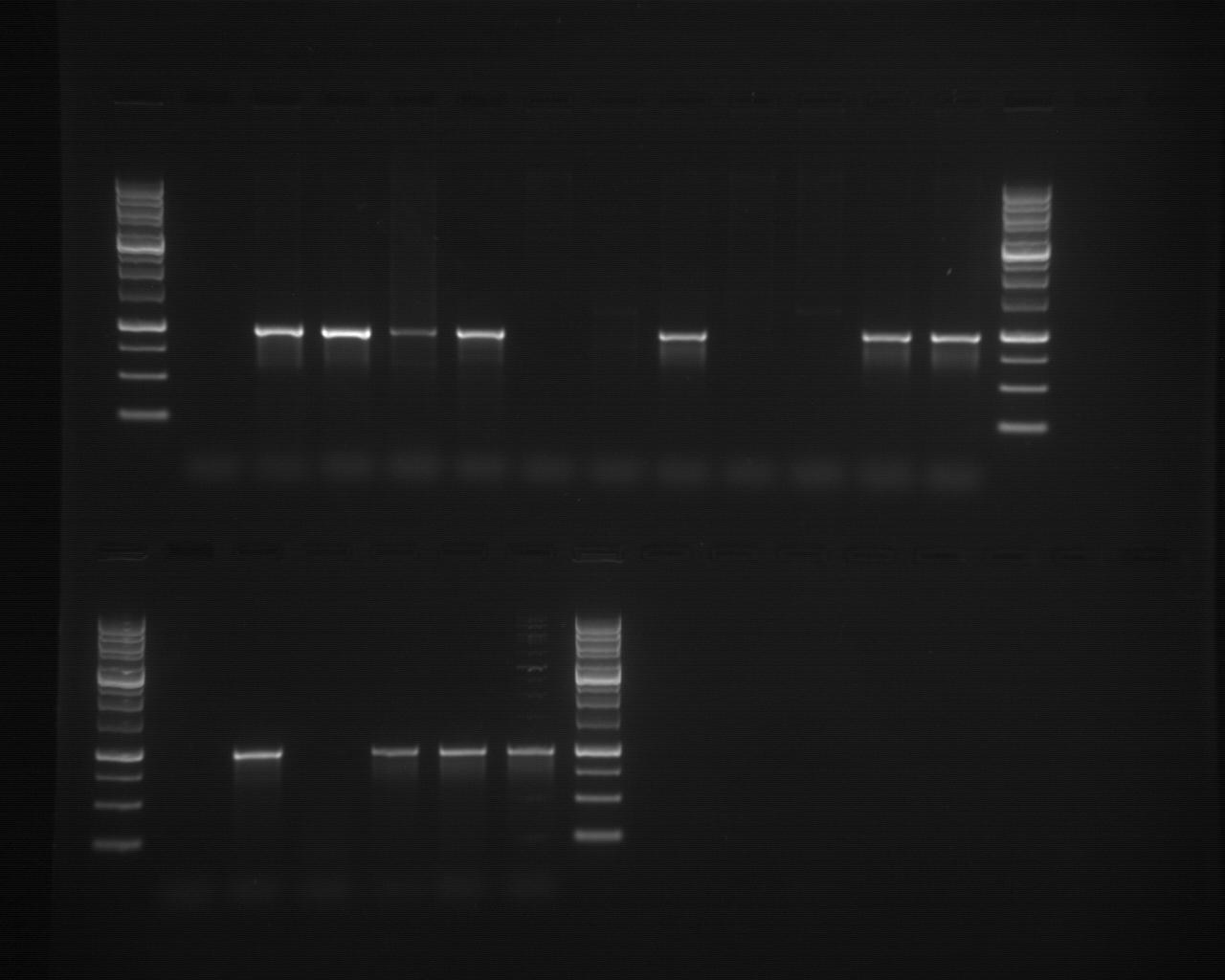

Supplement: Supplemental Information 4 [file peerj-11-16002-s004.zip › Figure 1/Target B/Target B.png]

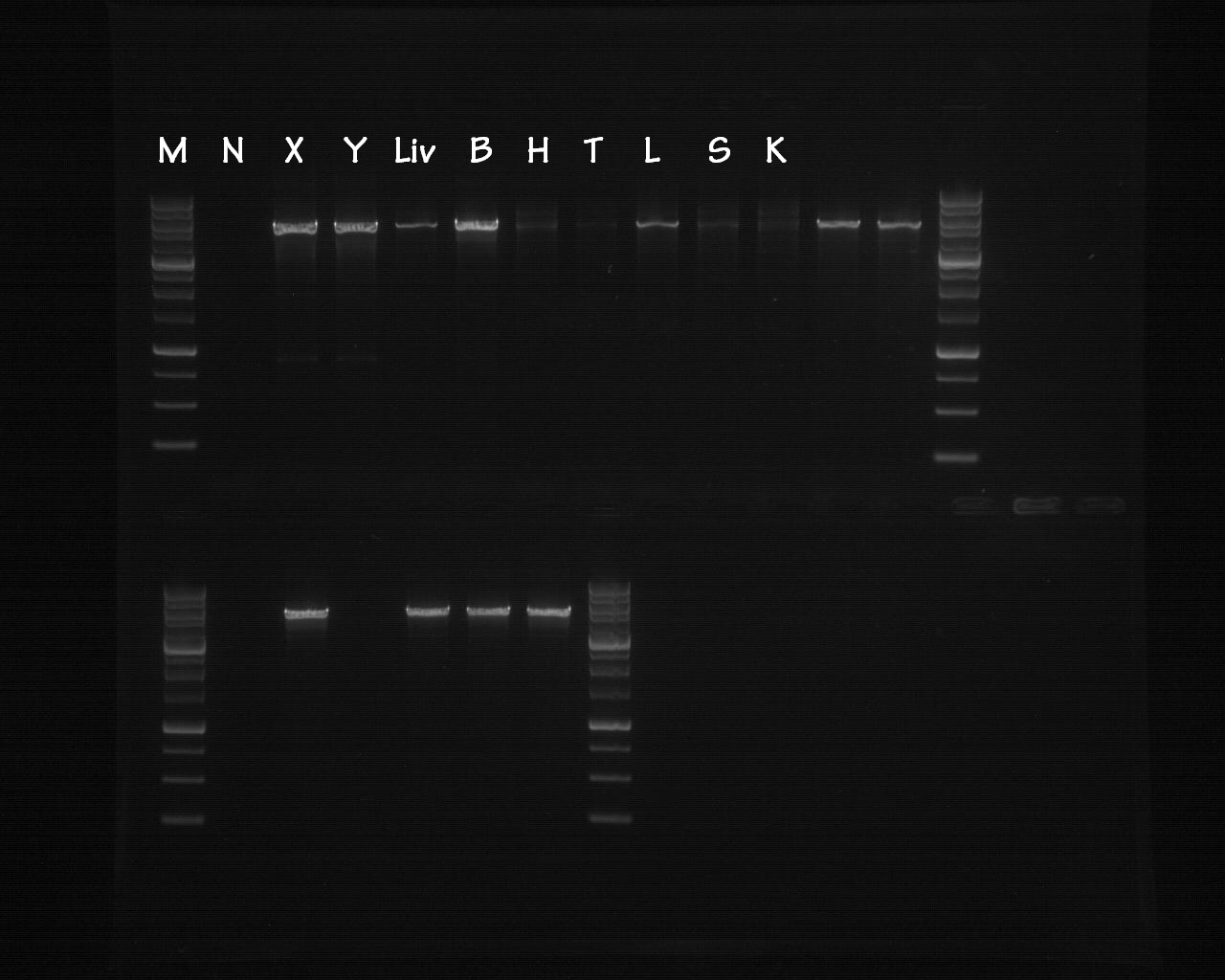

Supplement: Supplemental Information 4 [file peerj-11-16002-s004.zip › Figure 1/Target C/Target C labelled.png]

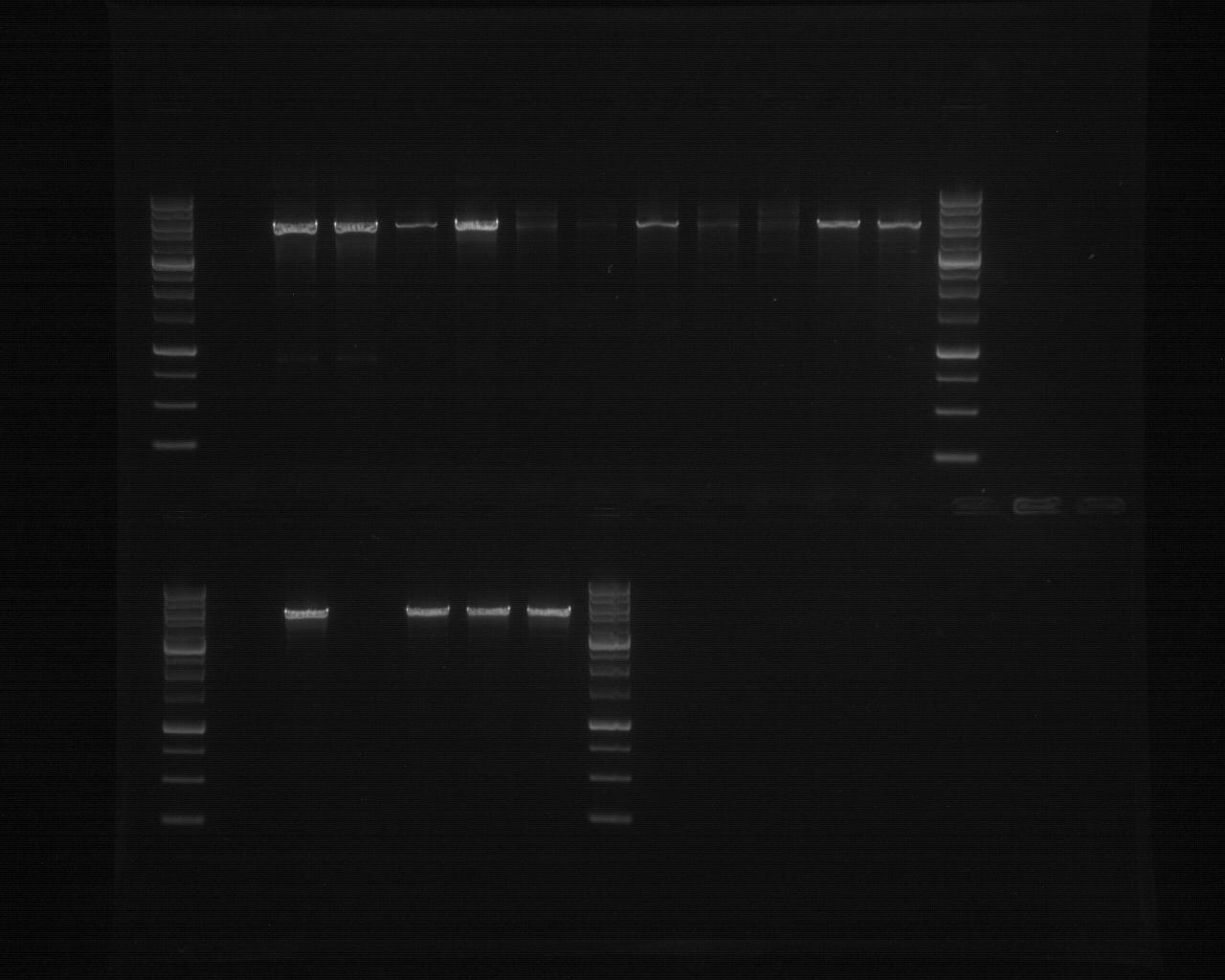

Supplement: Supplemental Information 4 [file peerj-11-16002-s004.zip › Figure 1/Target C/Target C.png]

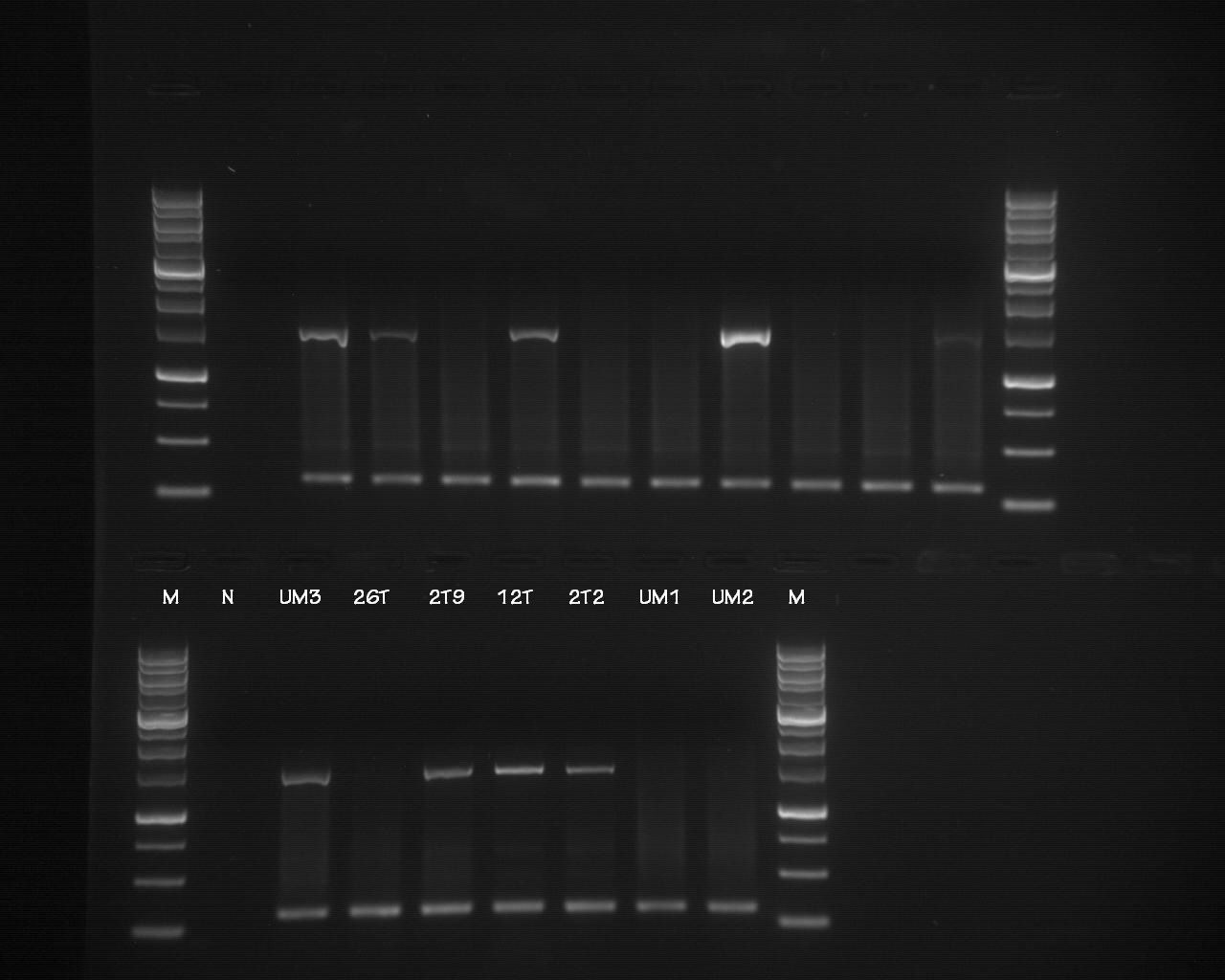

Supplement: Supplemental Information 5 [file peerj-11-16002-s005.zip › Figure 3/Target 16S/Target 16S labelled.jpg]

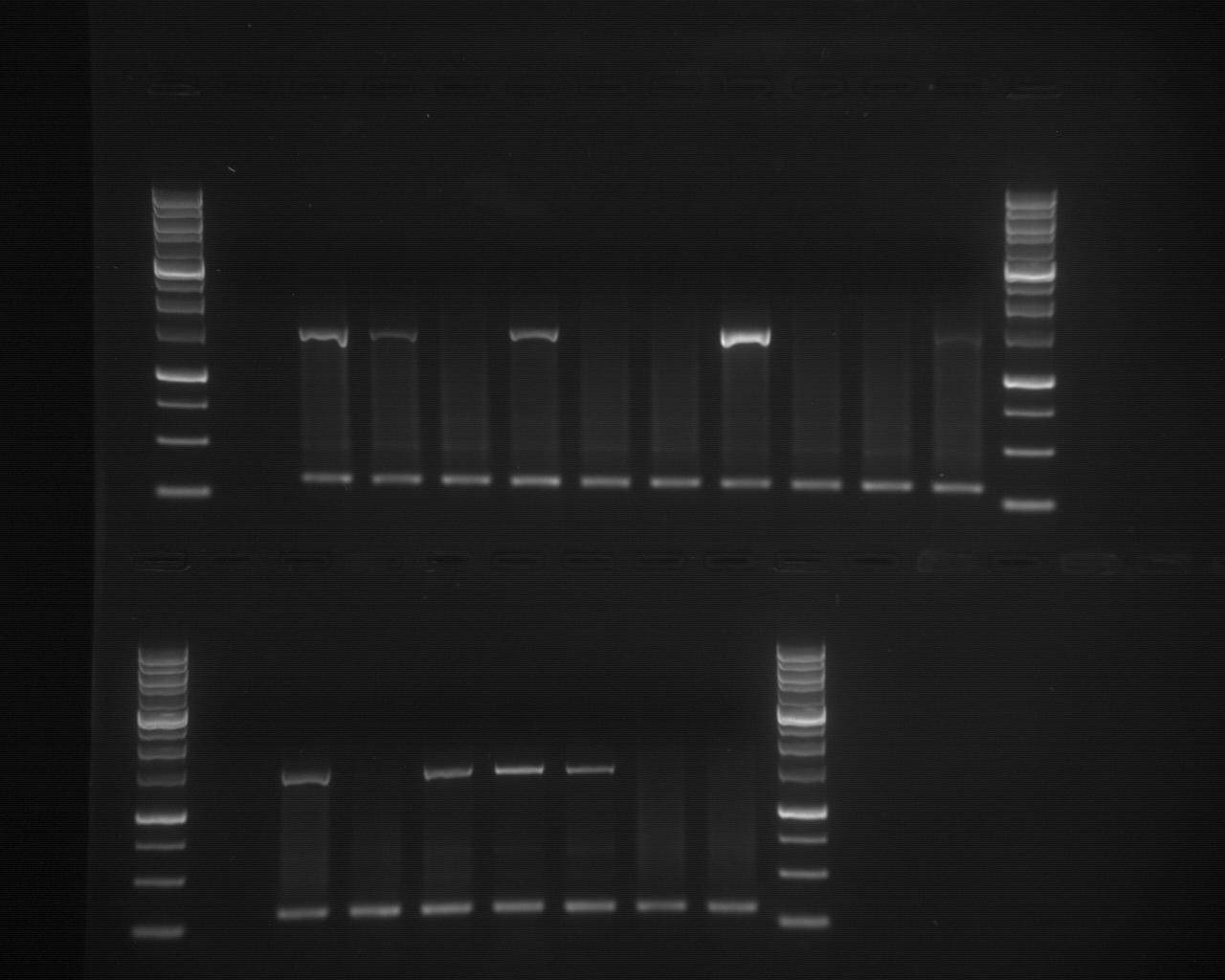

Supplement: Supplemental Information 5 [file peerj-11-16002-s005.zip › Figure 3/Target 16S/Target 16S.jpg]

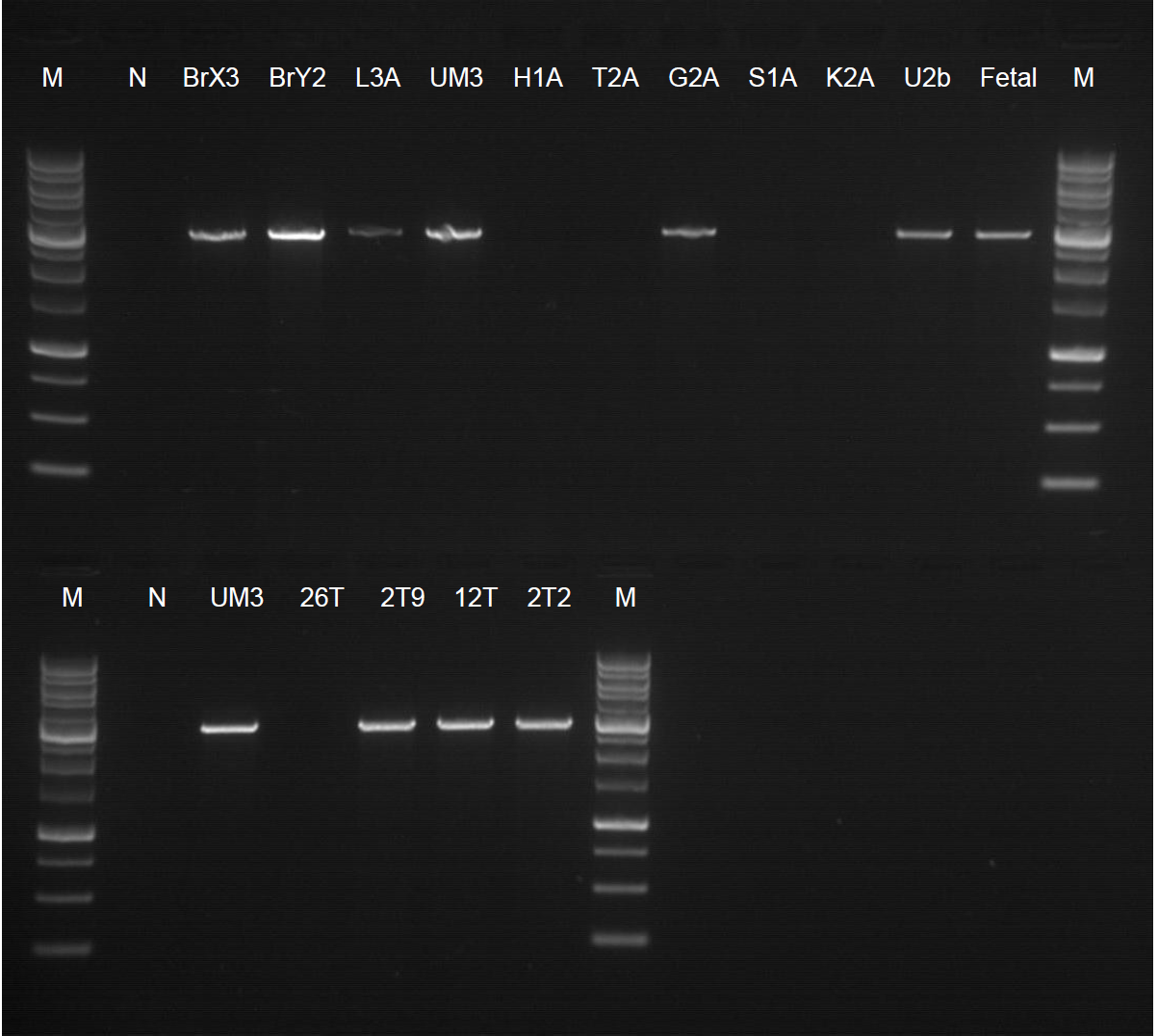

Supplement: Supplemental Information 5 [file peerj-11-16002-s005.zip › Figure 3/Target A/Target A left labelled.jpg]

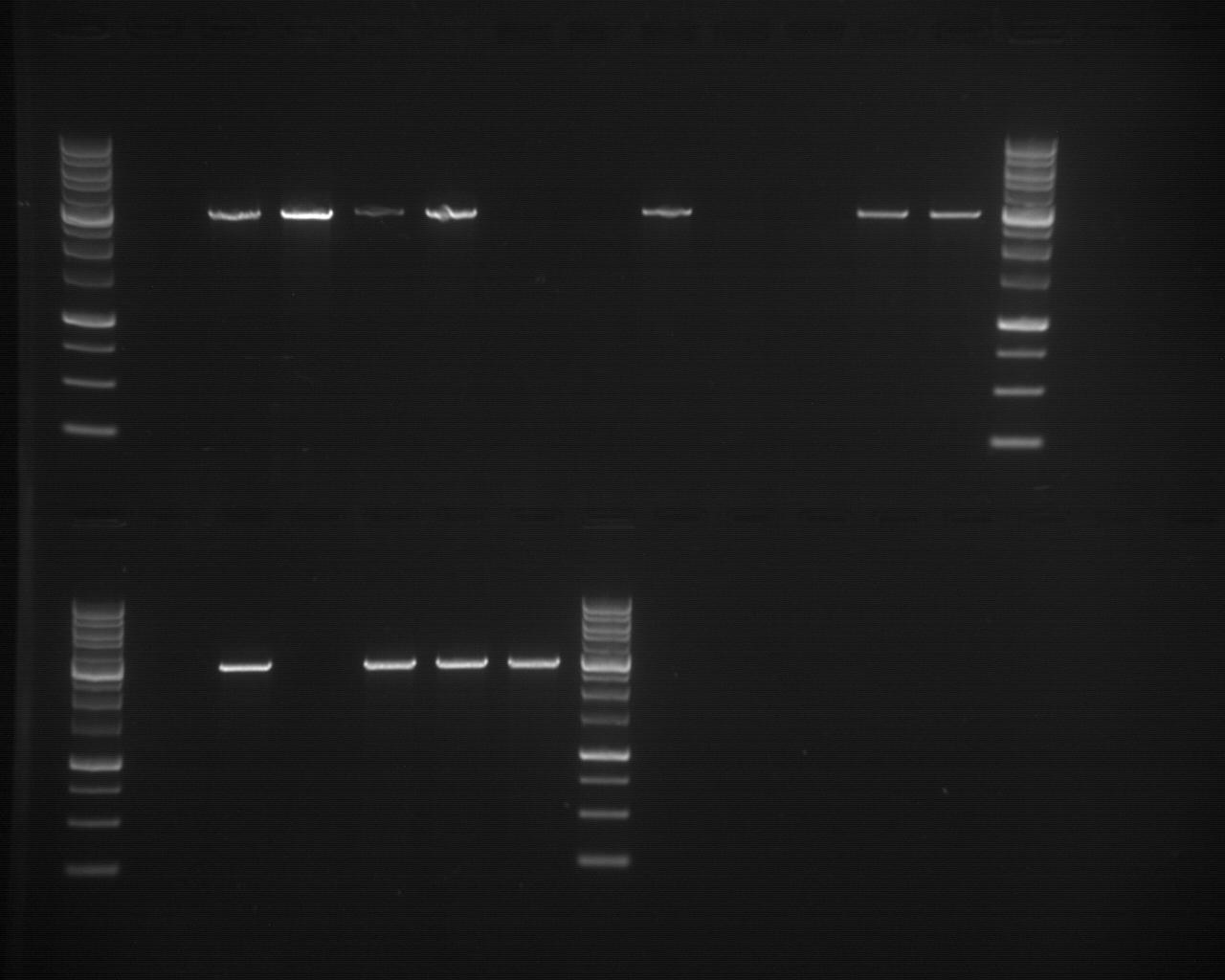

Supplement: Supplemental Information 5 [file peerj-11-16002-s005.zip › Figure 3/Target A/Target A left.jpg]

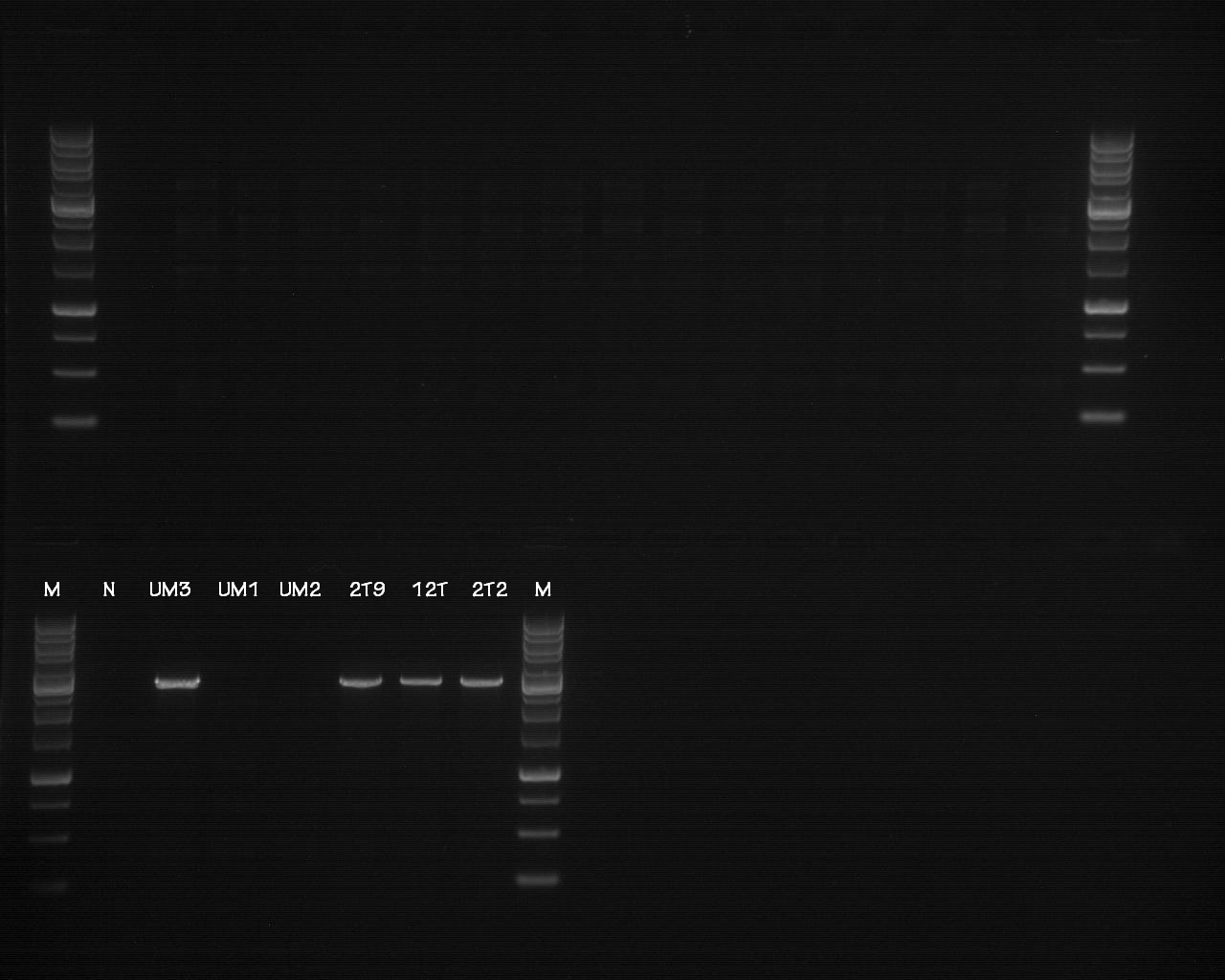

Supplement: Supplemental Information 5 [file peerj-11-16002-s005.zip › Figure 3/Target A/Target A right labelled.jpg]

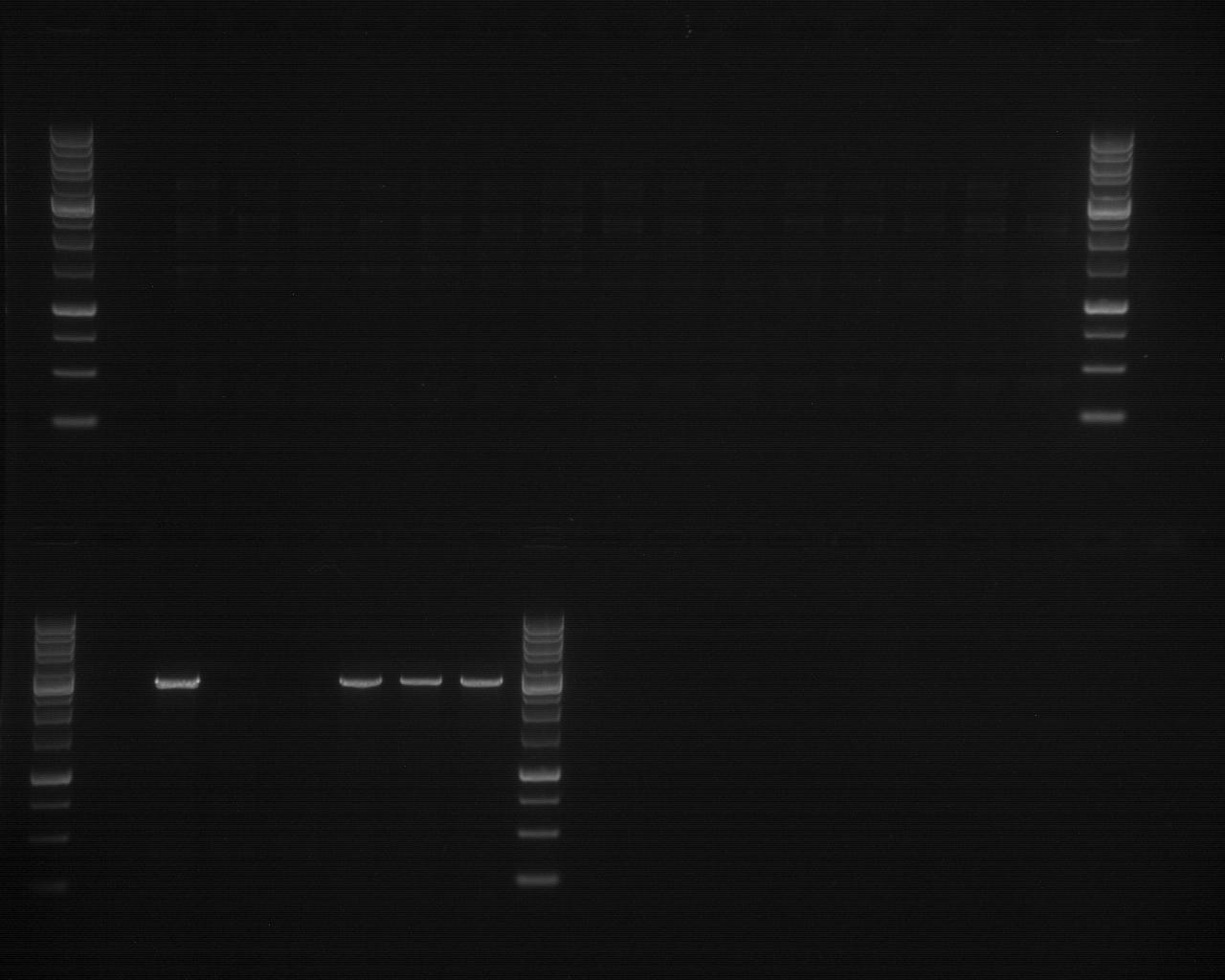

Supplement: Supplemental Information 5 [file peerj-11-16002-s005.zip › Figure 3/Target A/Target A right.jpg]

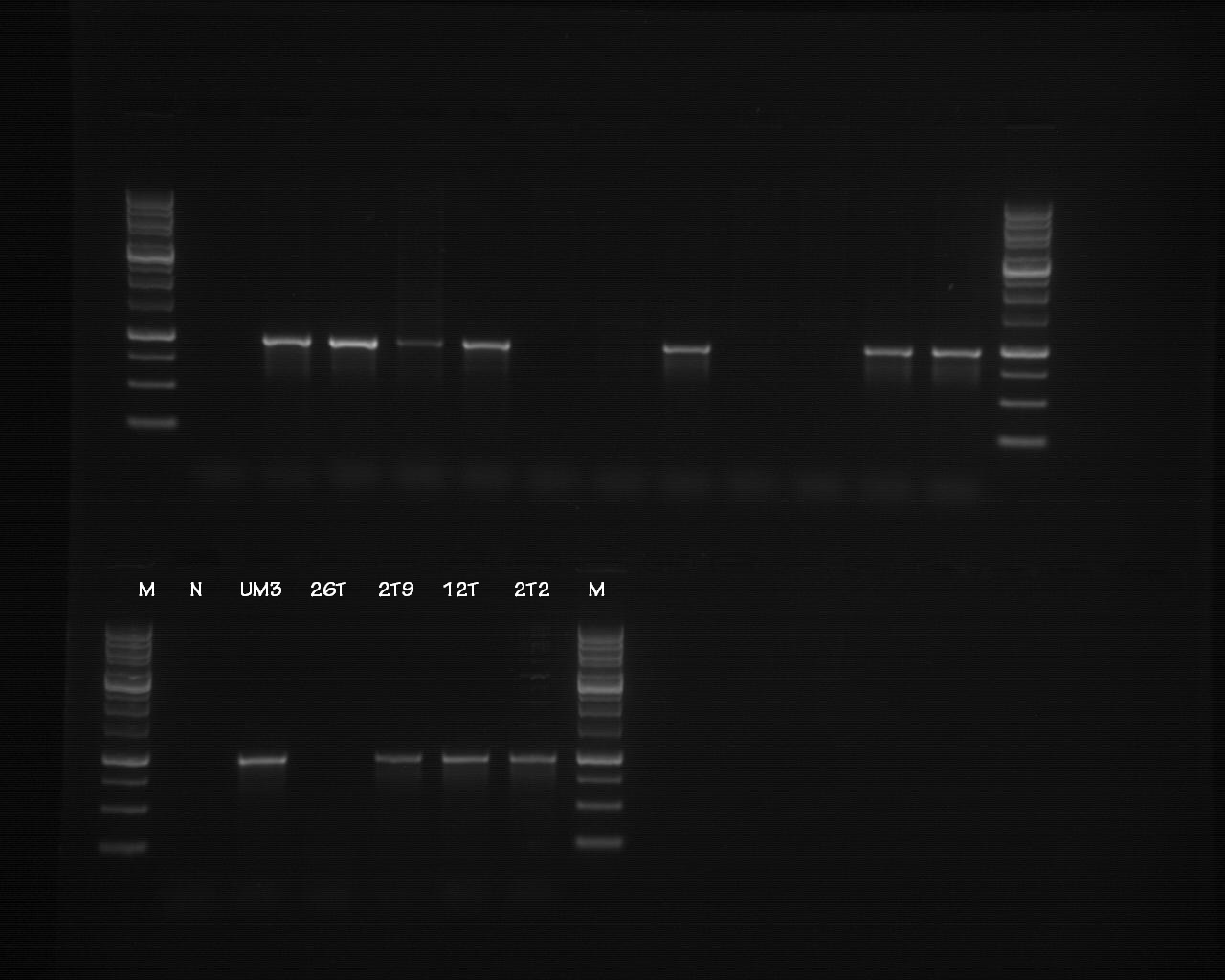

Supplement: Supplemental Information 5 [file peerj-11-16002-s005.zip › Figure 3/Target B/Target B left labelled.jpg]

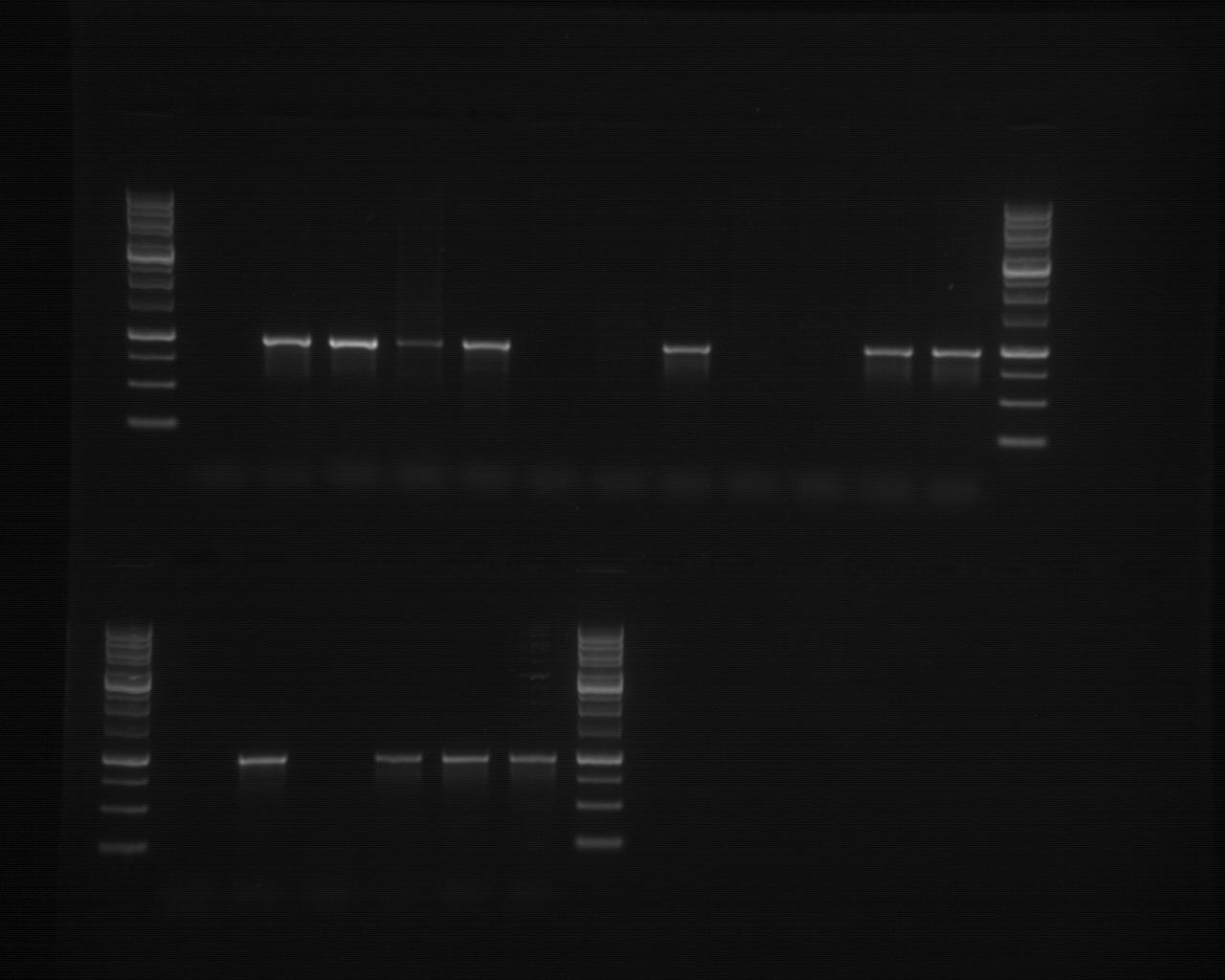

Supplement: Supplemental Information 5 [file peerj-11-16002-s005.zip › Figure 3/Target B/Target B left.jpg]

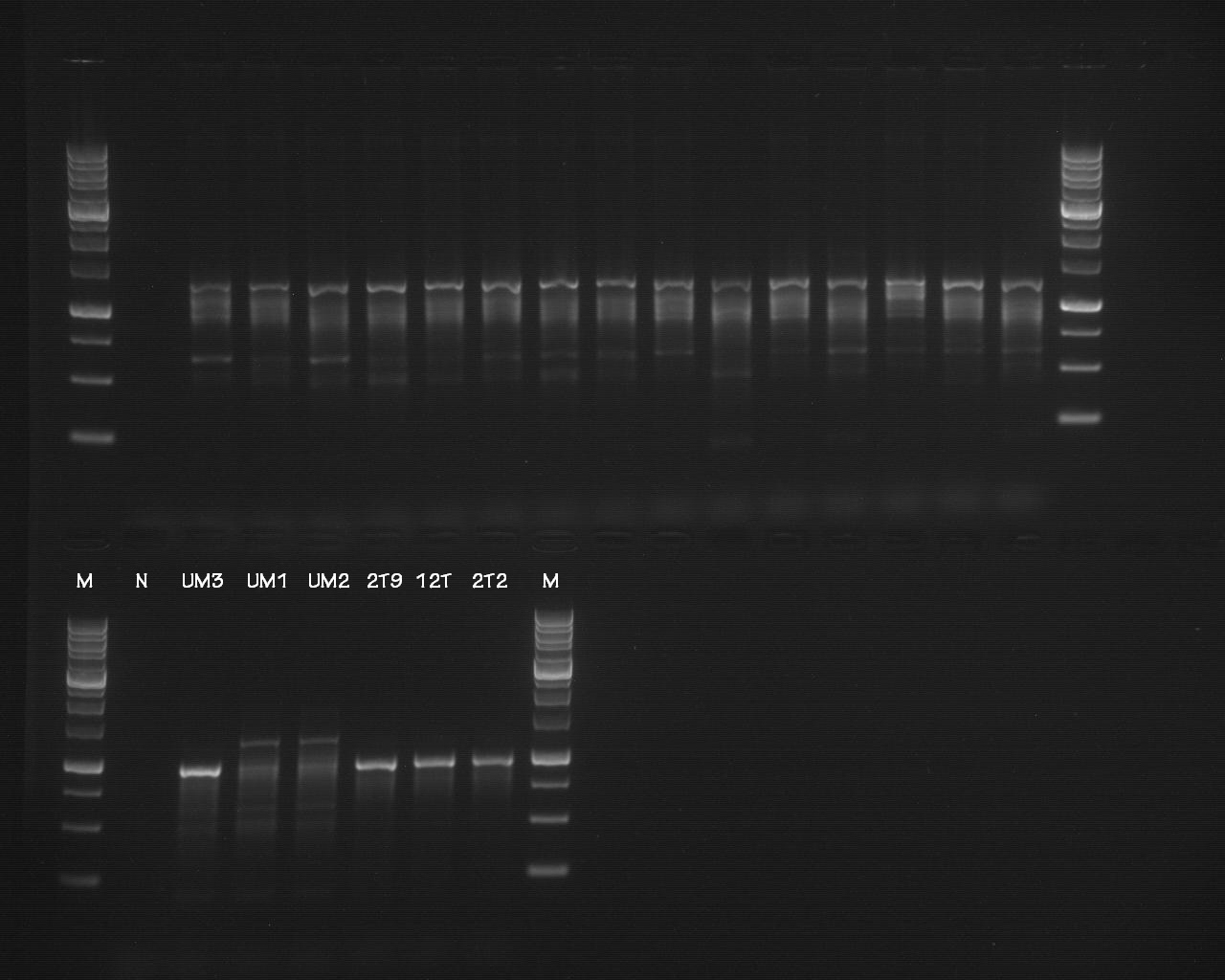

Supplement: Supplemental Information 5 [file peerj-11-16002-s005.zip › Figure 3/Target B/Target B right labelled.jpg]

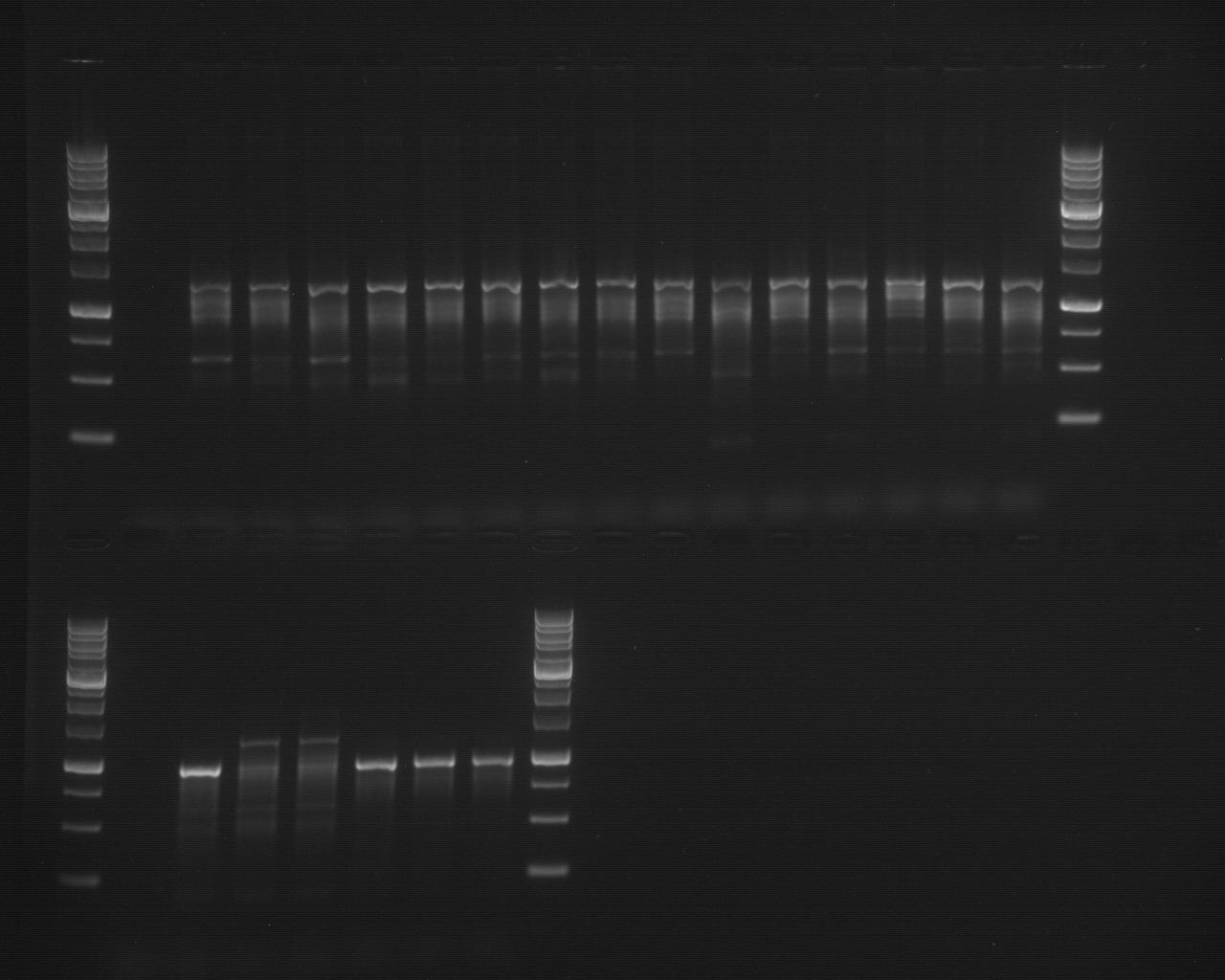

Supplement: Supplemental Information 5 [file peerj-11-16002-s005.zip › Figure 3/Target B/Target B right.jpg]

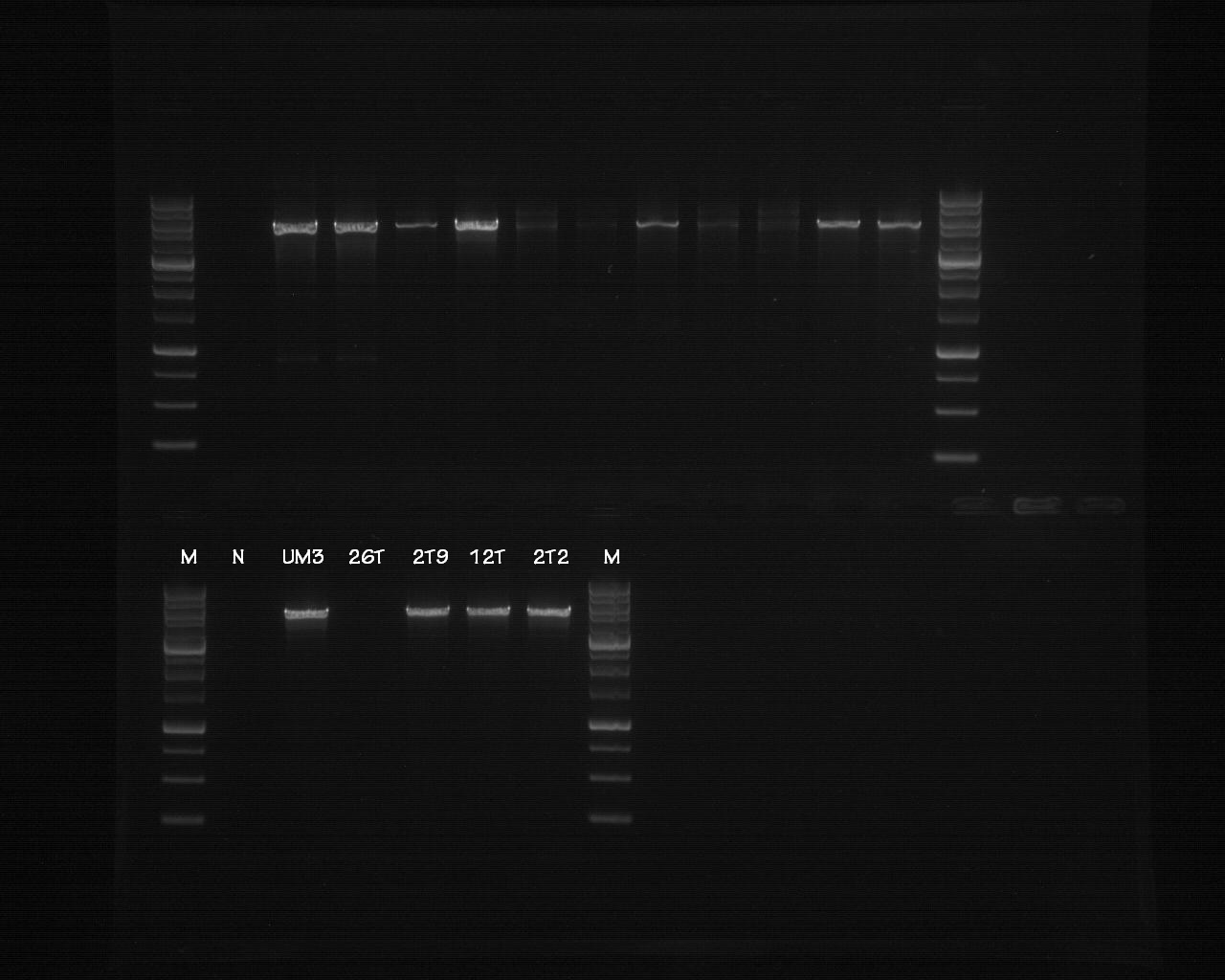

Supplement: Supplemental Information 5 [file peerj-11-16002-s005.zip › Figure 3/Target C/Target C left labelled.jpg]

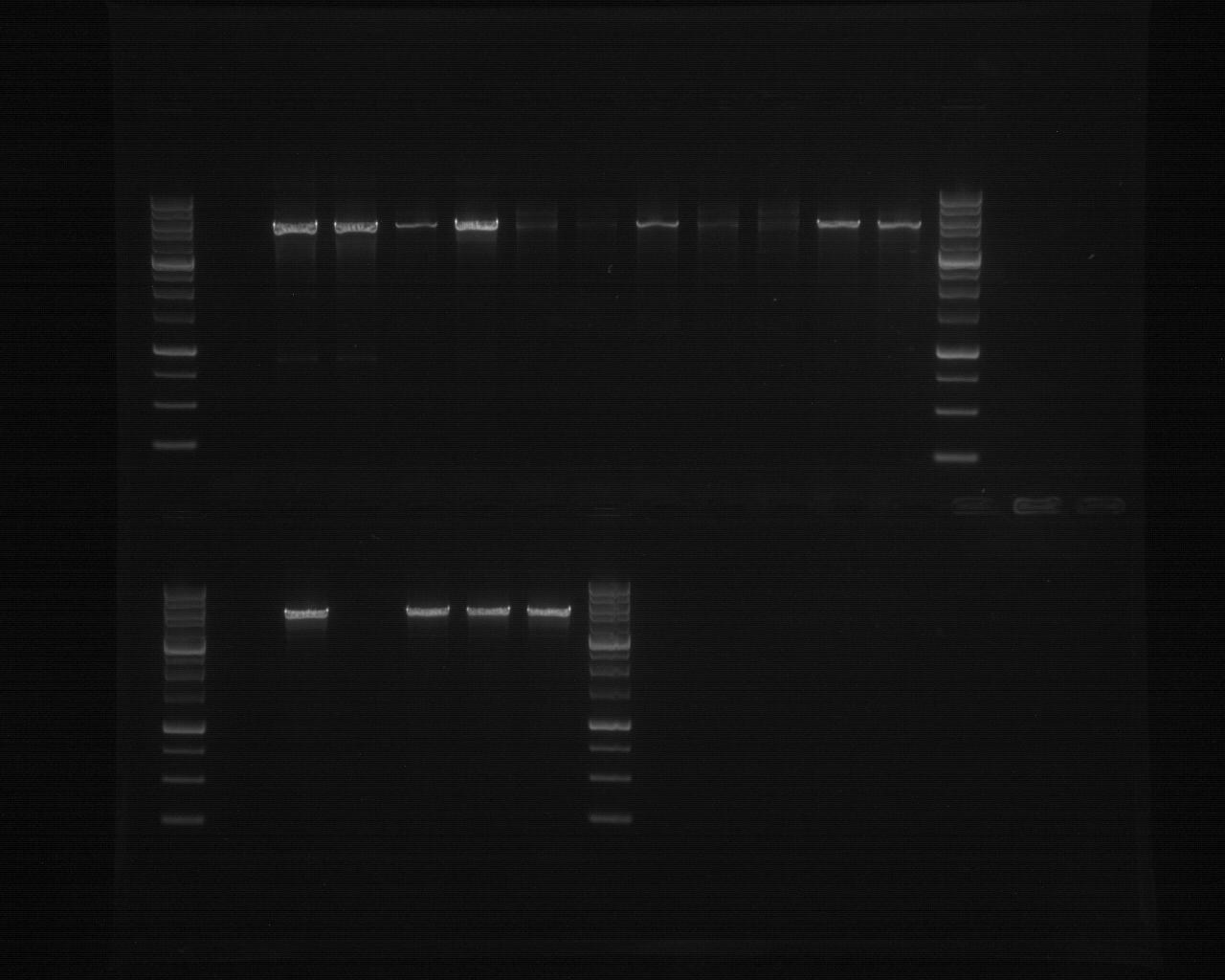

Supplement: Supplemental Information 5 [file peerj-11-16002-s005.zip › Figure 3/Target C/Target C left.jpg]

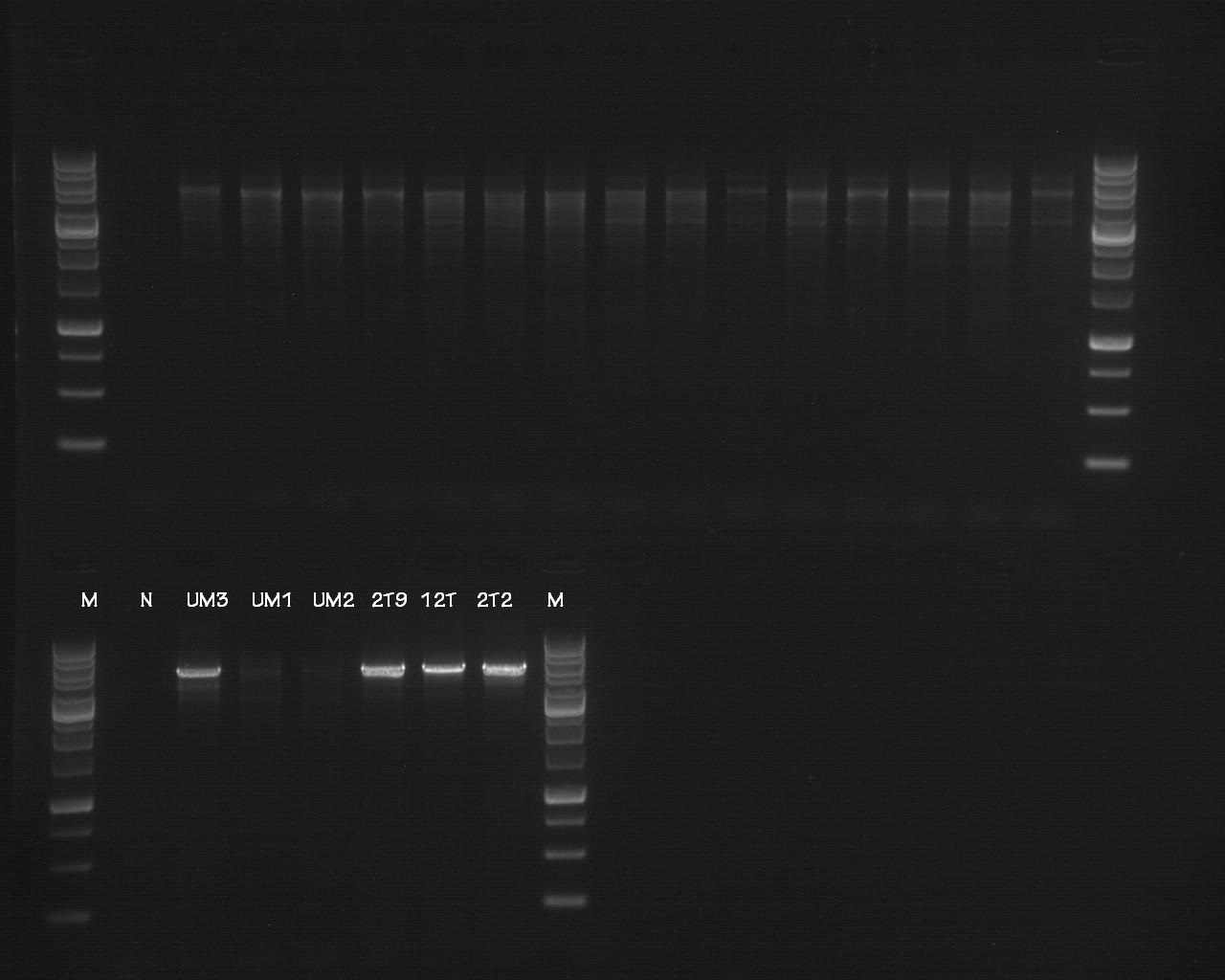

Supplement: Supplemental Information 5 [file peerj-11-16002-s005.zip › Figure 3/Target C/Target C right labelled.jpg]

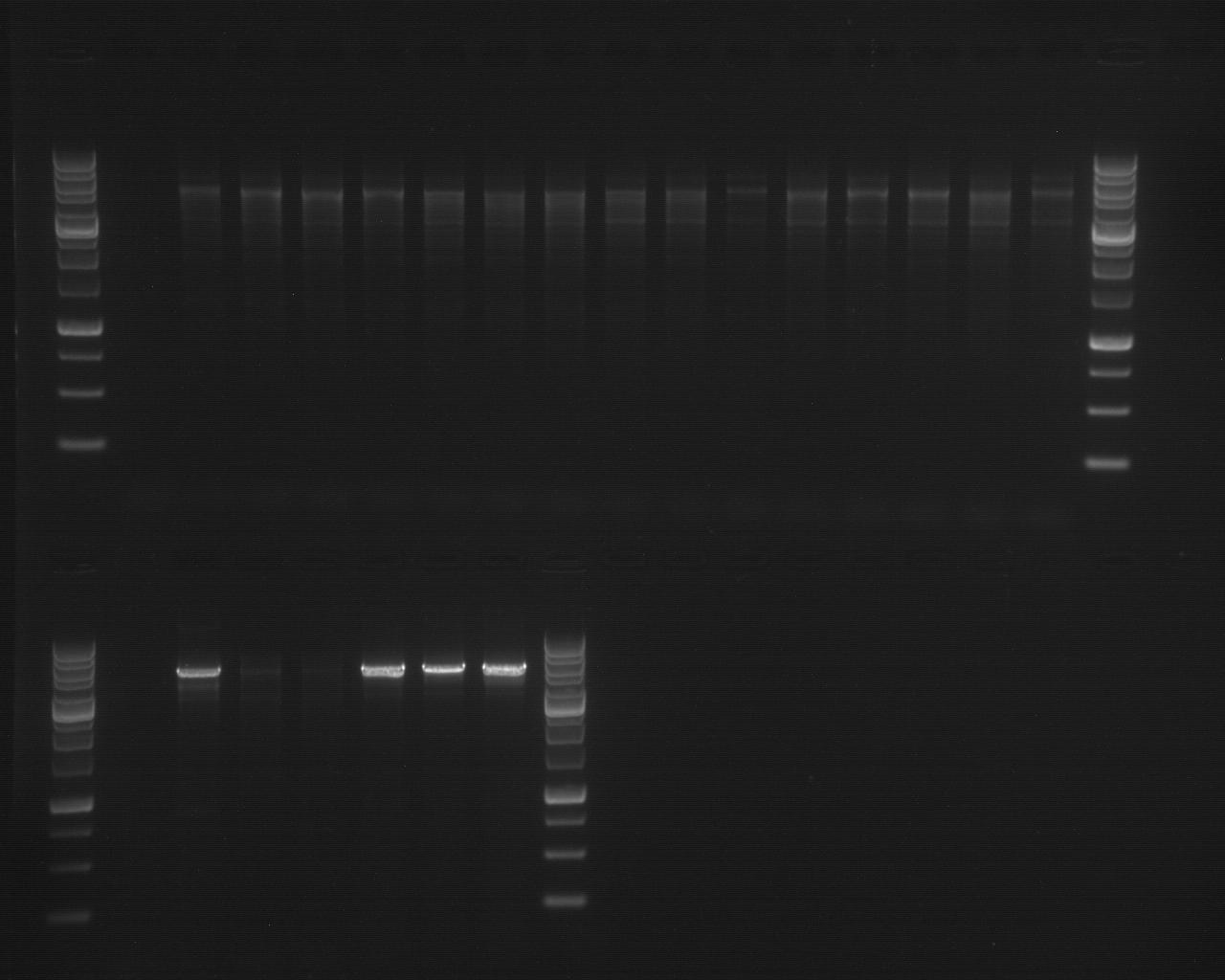

Supplement: Supplemental Information 5 [file peerj-11-16002-s005.zip › Figure 3/Target C/Target C right.jpg]

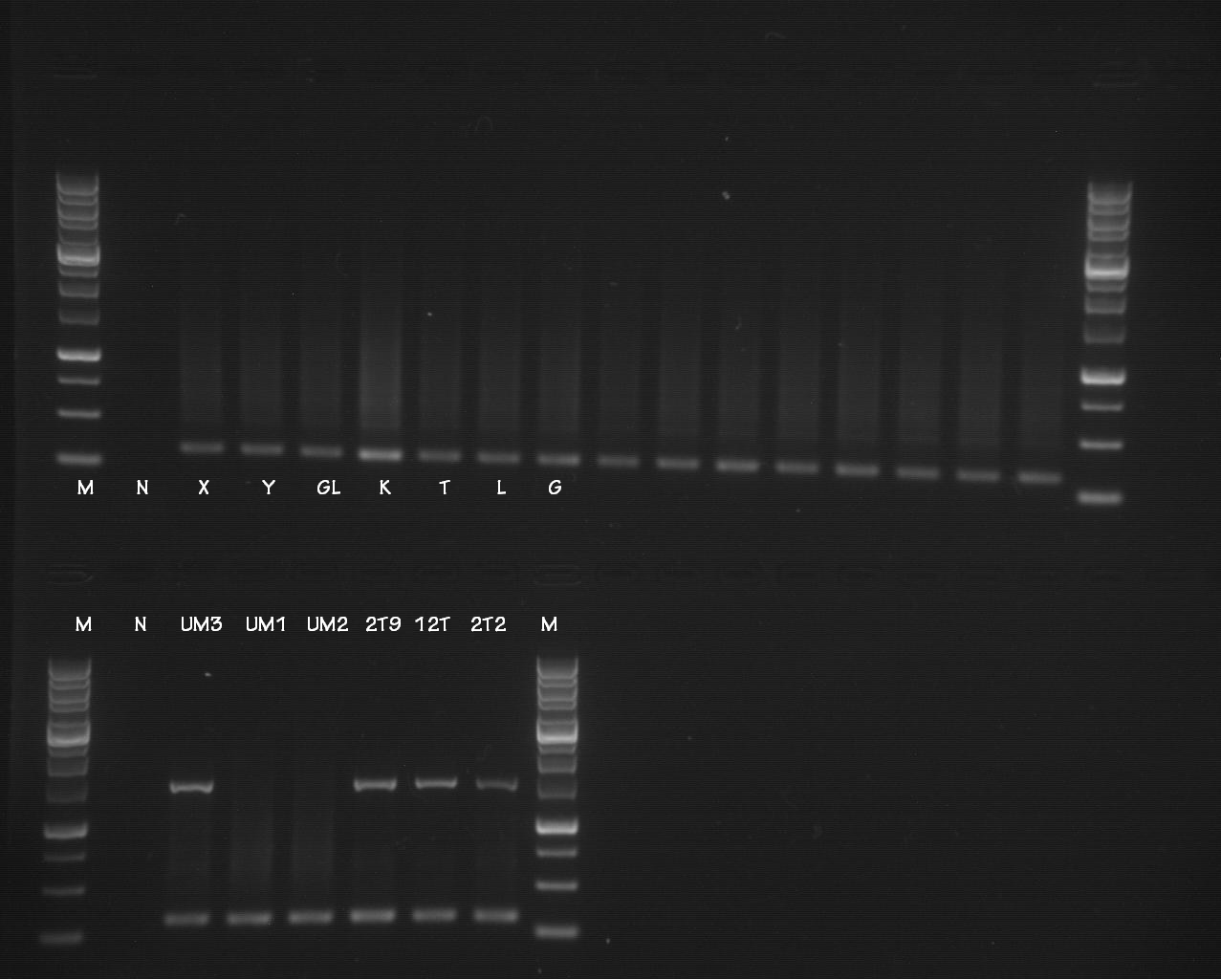

Supplement: Supplemental Information 6 [file peerj-11-16002-s006.zip › Figure 4/Target 16S/Target 16S labelled.png]

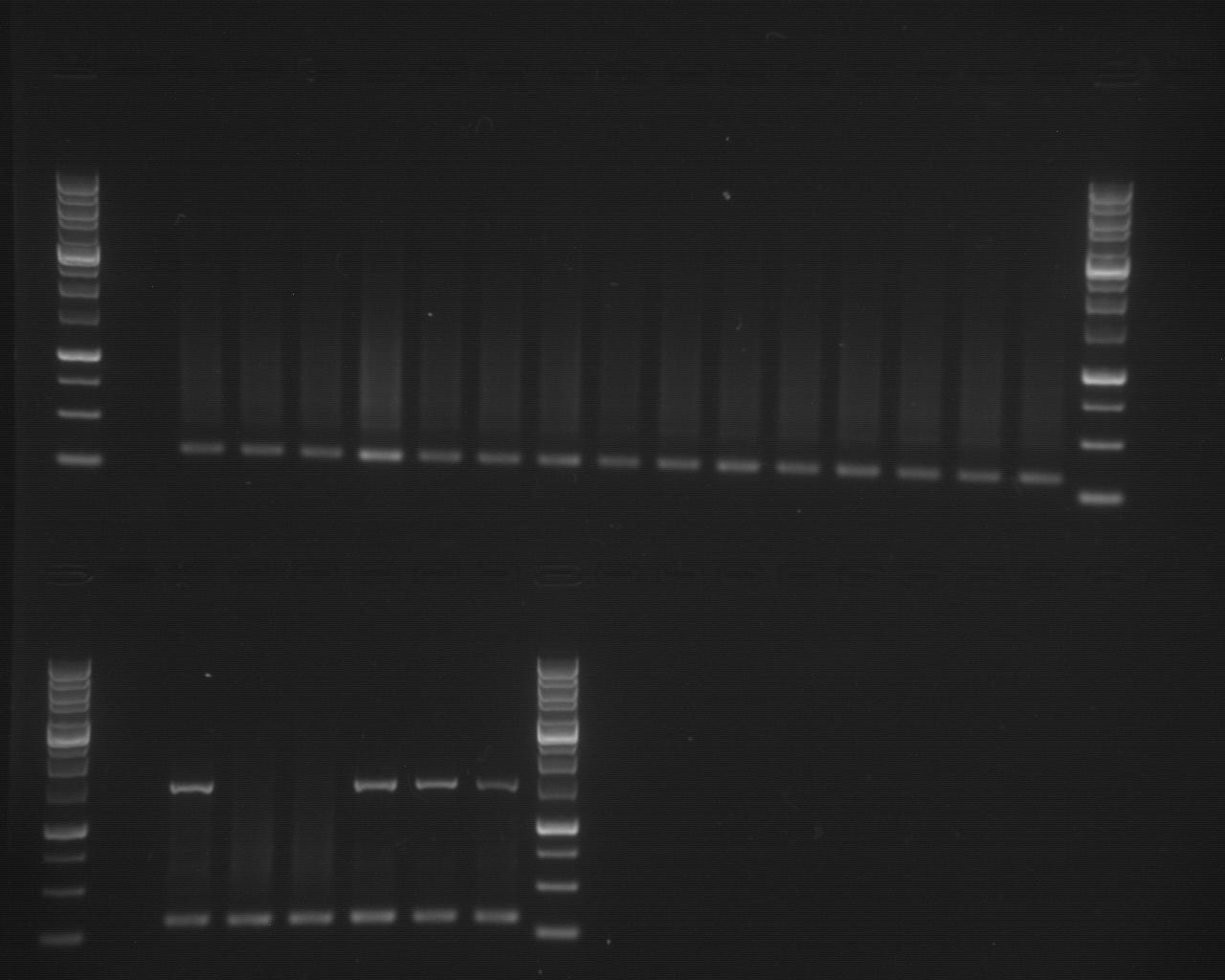

Supplement: Supplemental Information 6 [file peerj-11-16002-s006.zip › Figure 4/Target 16S/Target 16S.png]

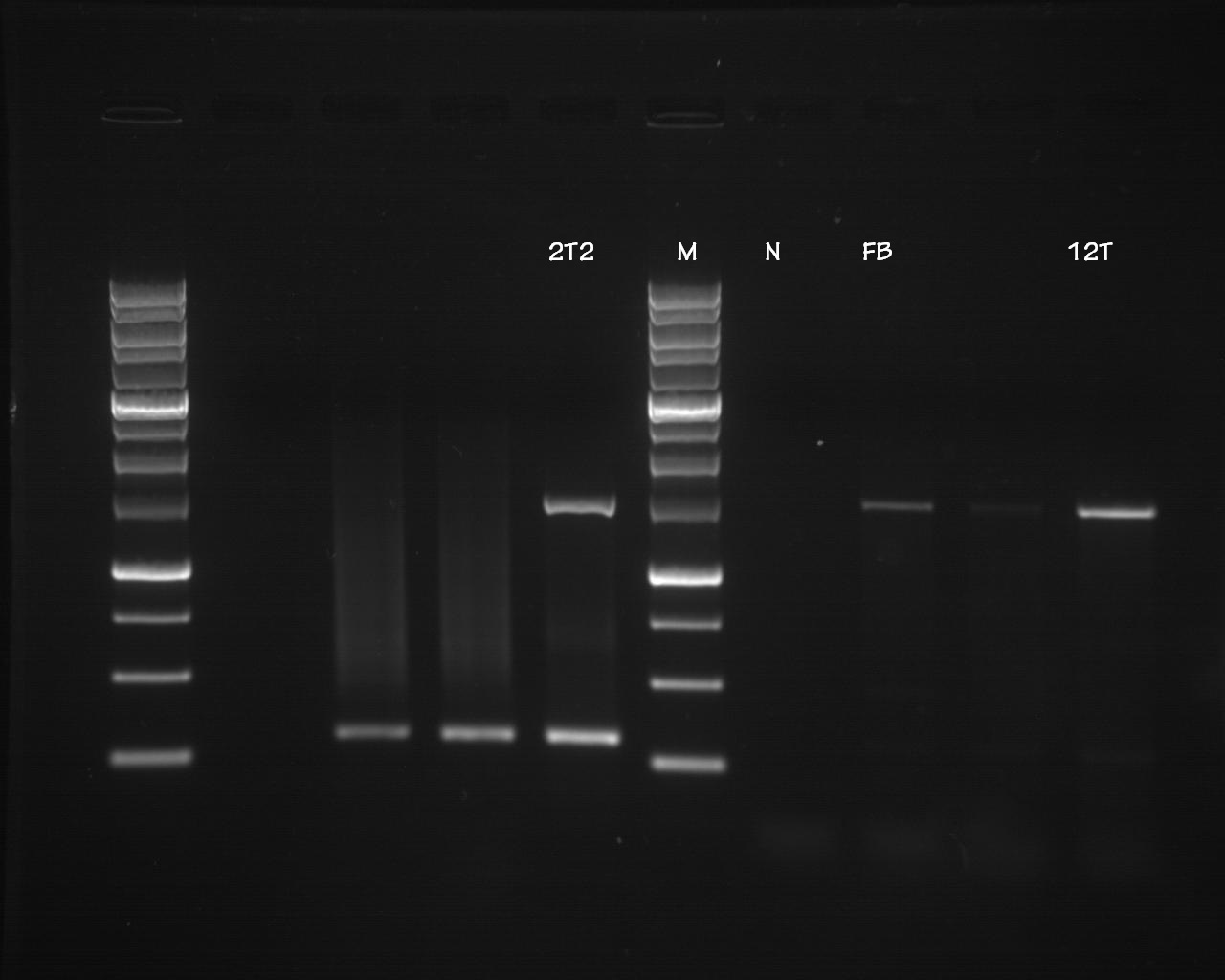

Supplement: Supplemental Information 6 [file peerj-11-16002-s006.zip › Figure 4/Target 16S/Target 16S_FB labelled.jpg]

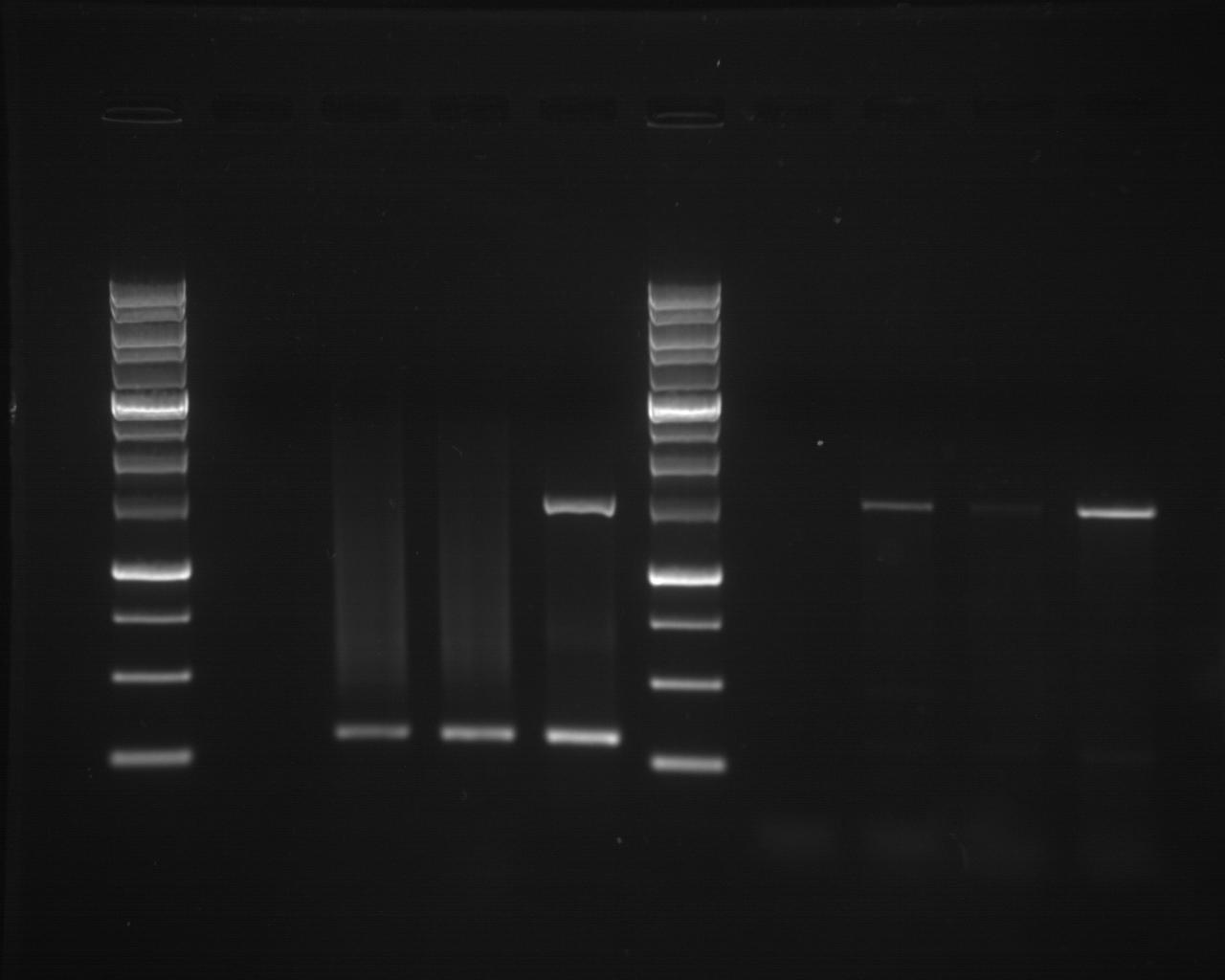

Supplement: Supplemental Information 6 [file peerj-11-16002-s006.zip › Figure 4/Target 16S/Target 16S_FB.jpg]

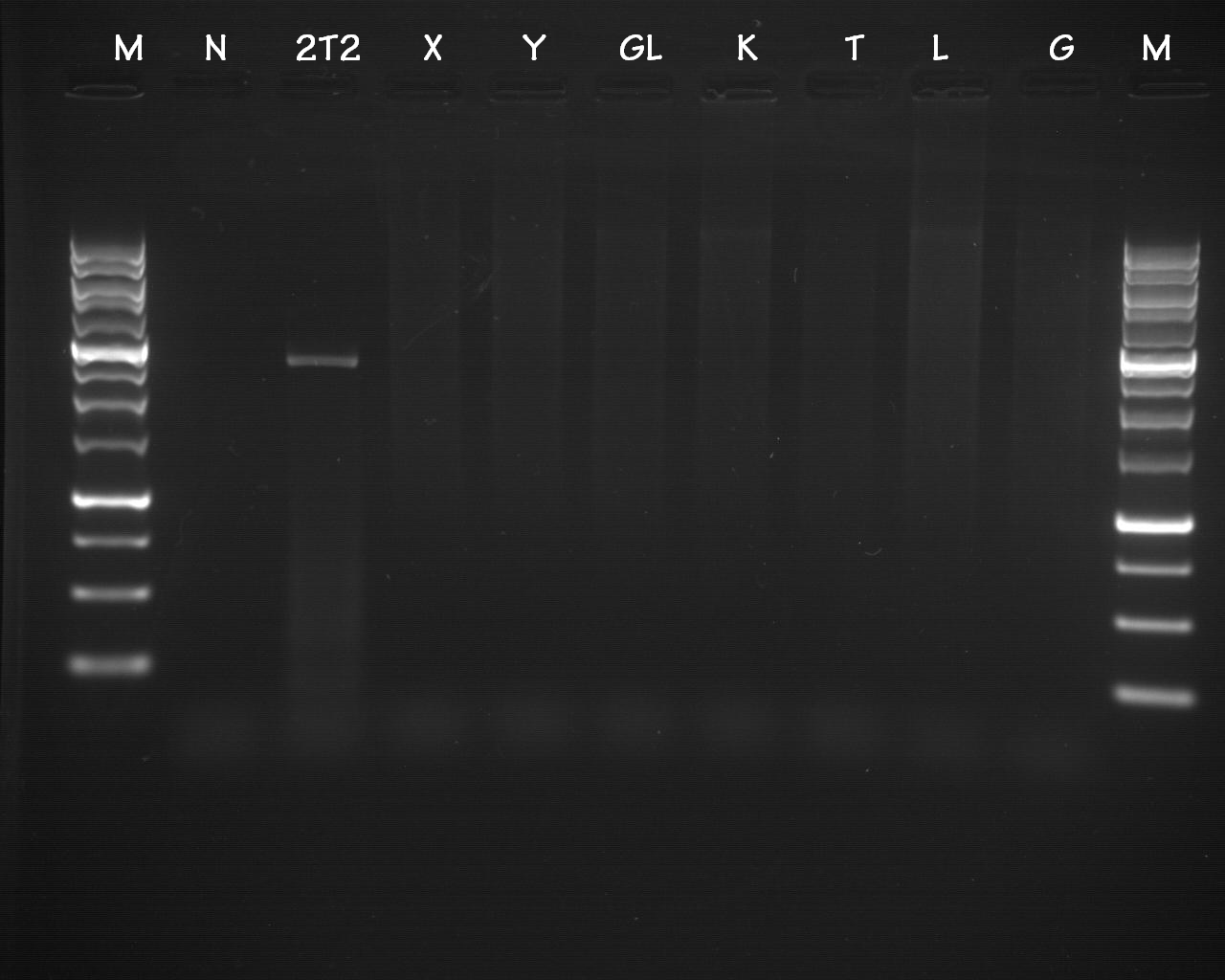

Supplement: Supplemental Information 6 [file peerj-11-16002-s006.zip › Figure 4/Target A/Target A labelled.png]

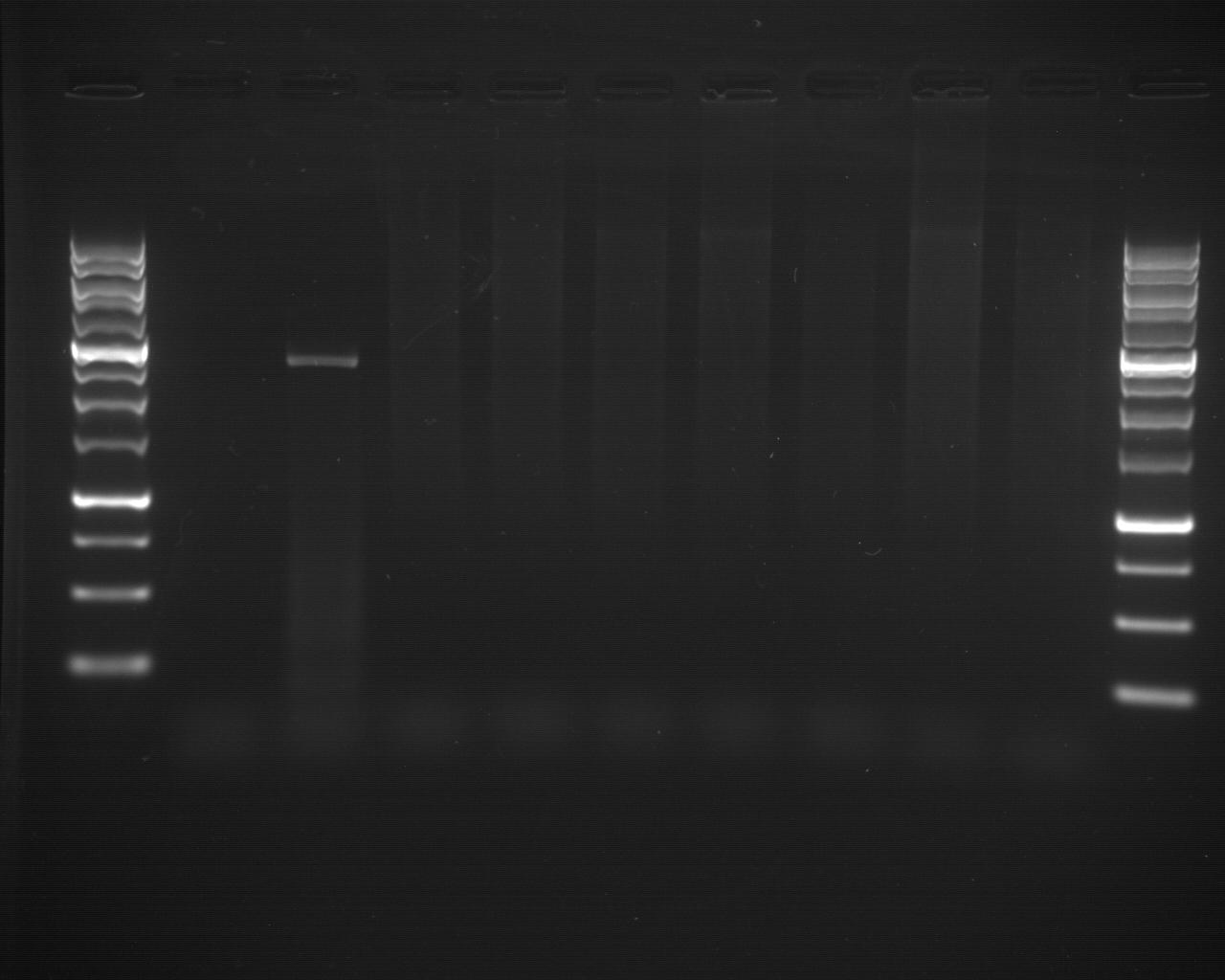

Supplement: Supplemental Information 6 [file peerj-11-16002-s006.zip › Figure 4/Target A/Target A.png]

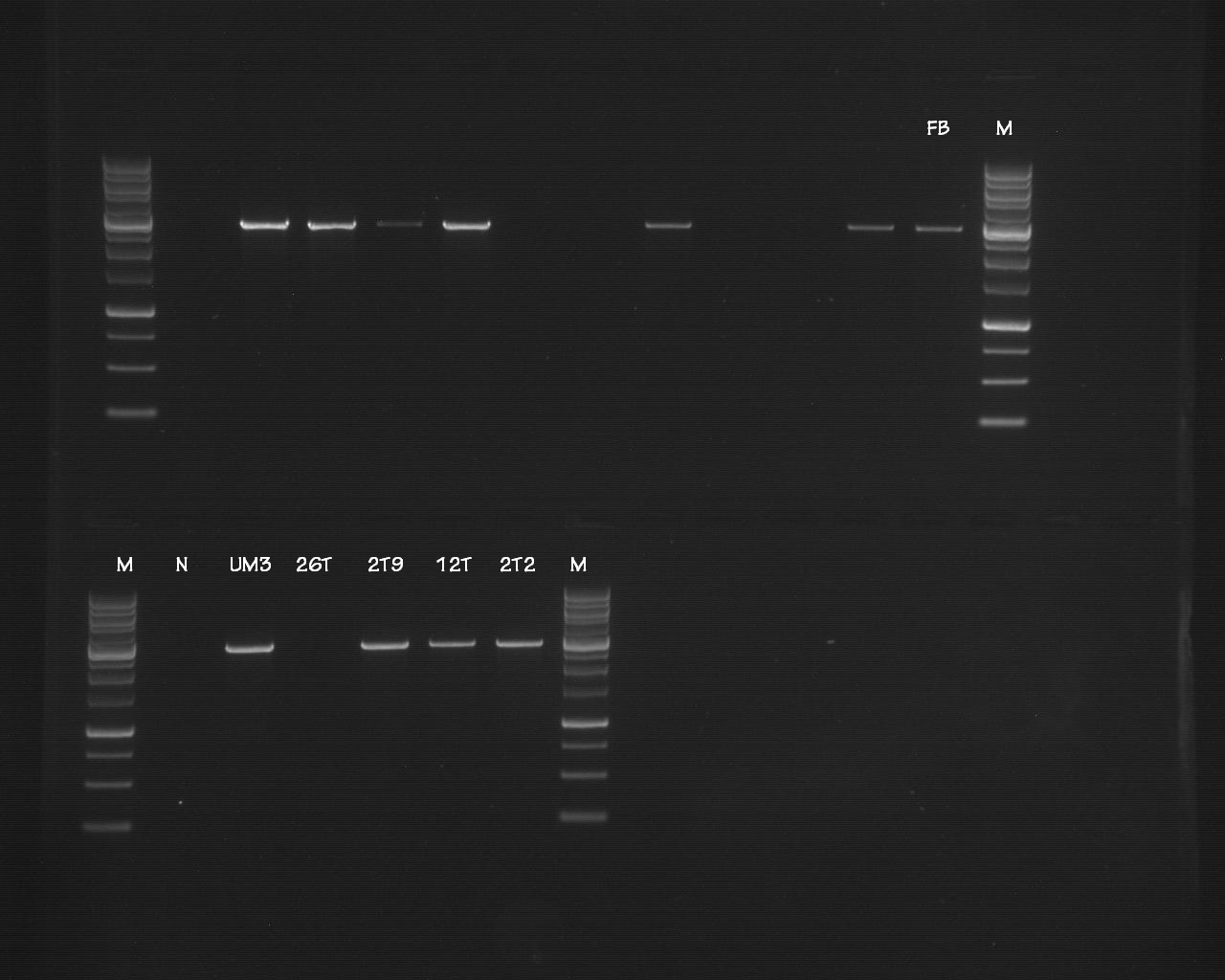

Supplement: Supplemental Information 6 [file peerj-11-16002-s006.zip › Figure 4/Target A/Target A_FB labelled.png]

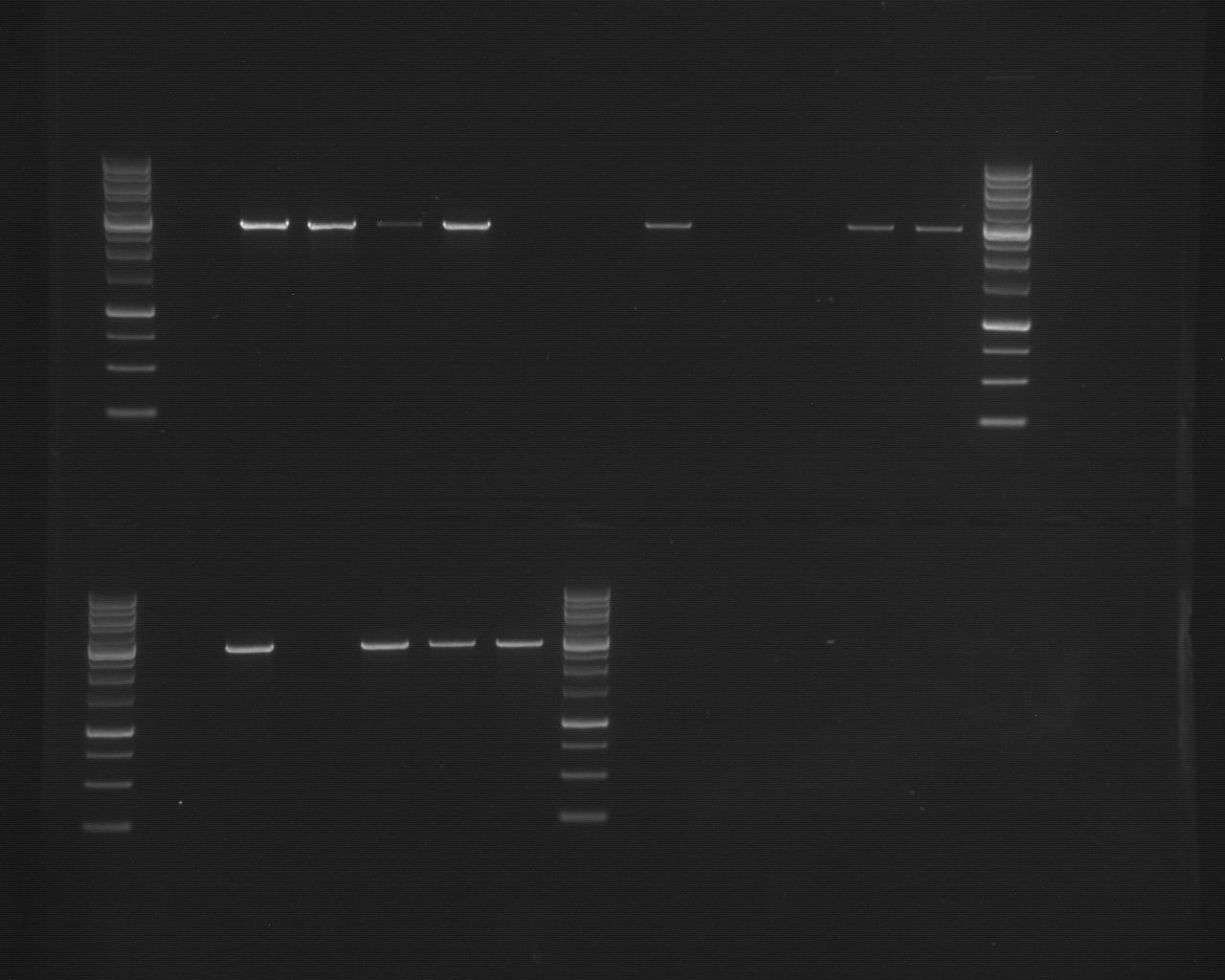

Supplement: Supplemental Information 6 [file peerj-11-16002-s006.zip › Figure 4/Target A/Target A_FB.png]

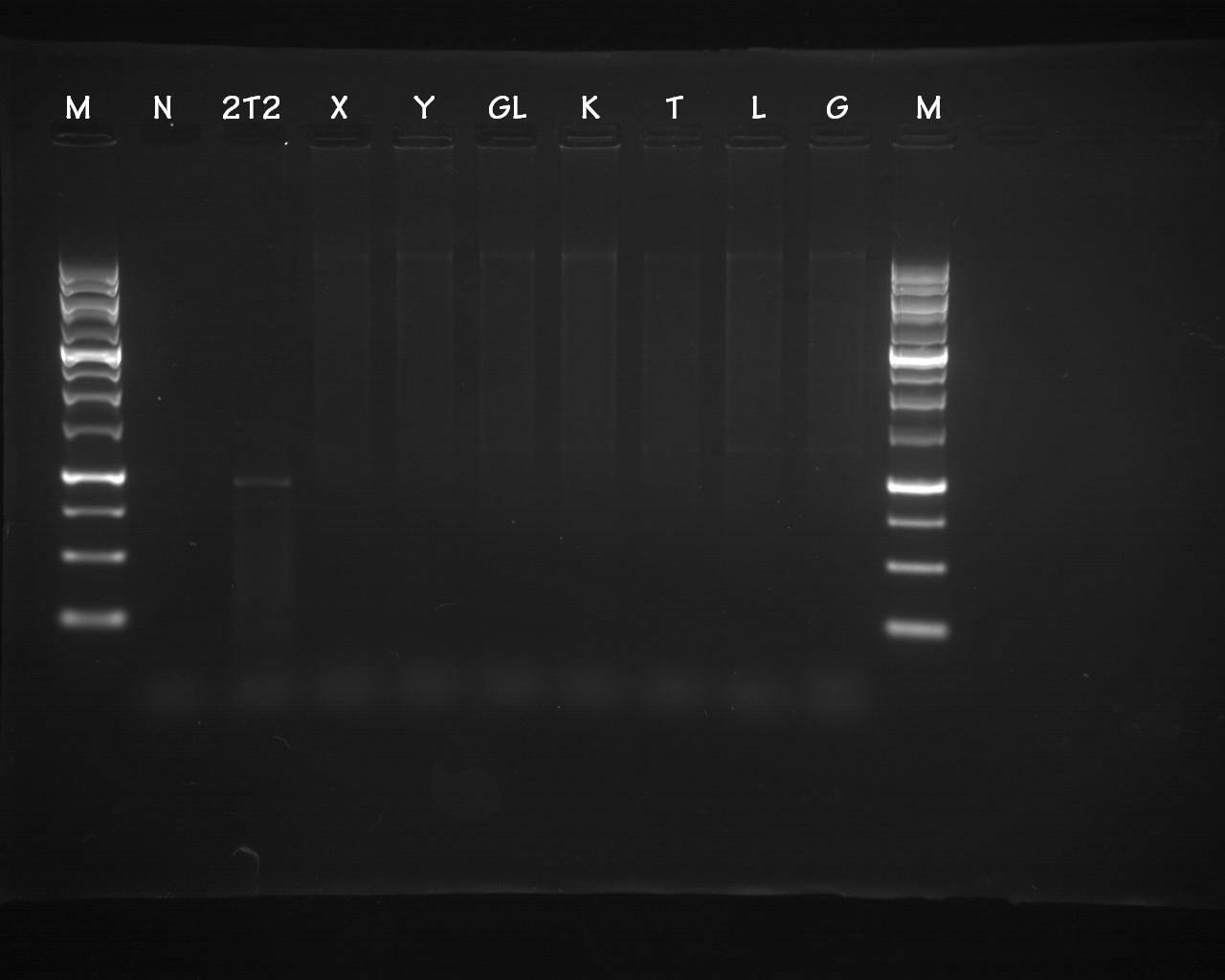

Supplement: Supplemental Information 6 [file peerj-11-16002-s006.zip › Figure 4/Target B/Target B labelled.png]

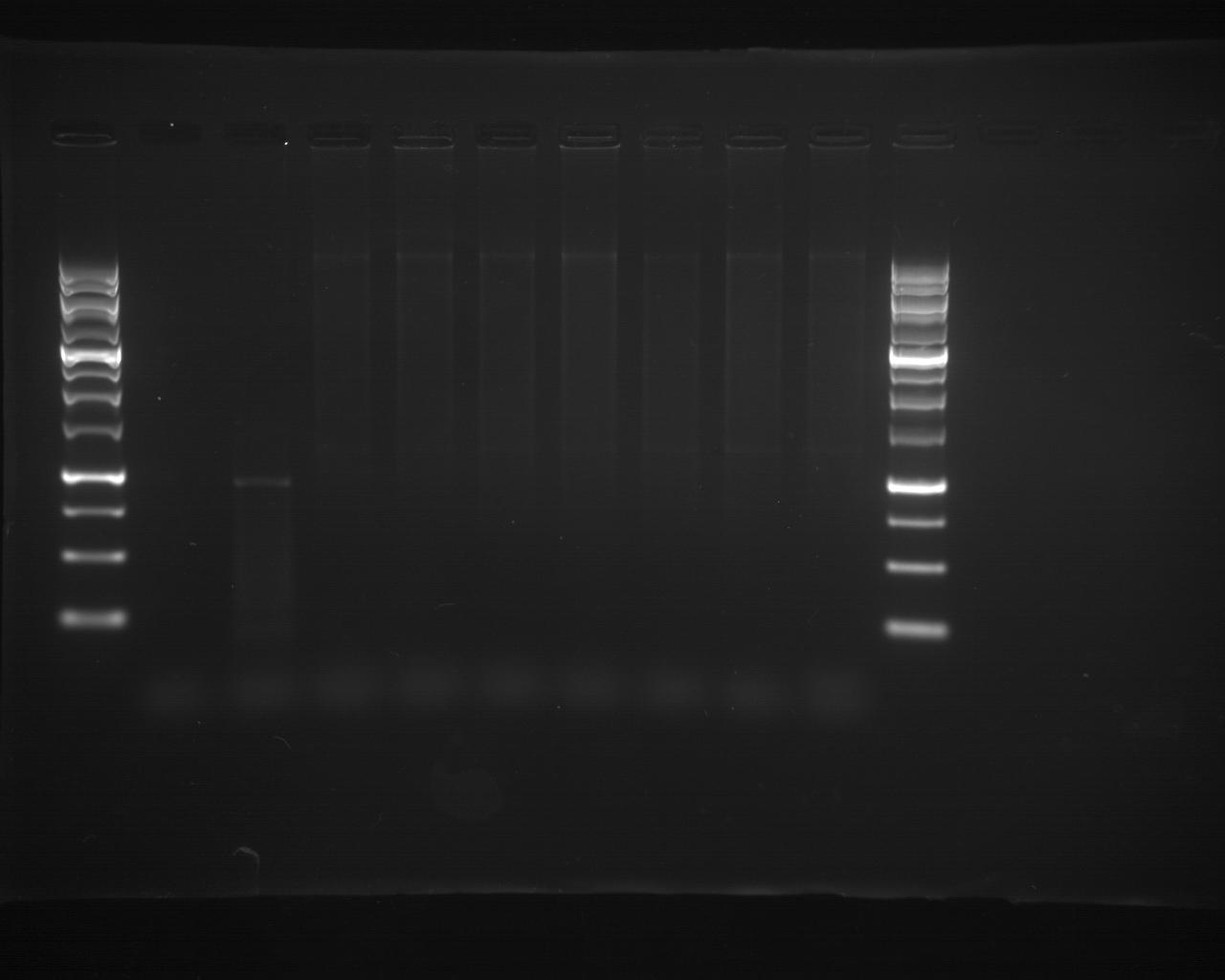

Supplement: Supplemental Information 6 [file peerj-11-16002-s006.zip › Figure 4/Target B/Target B.png]

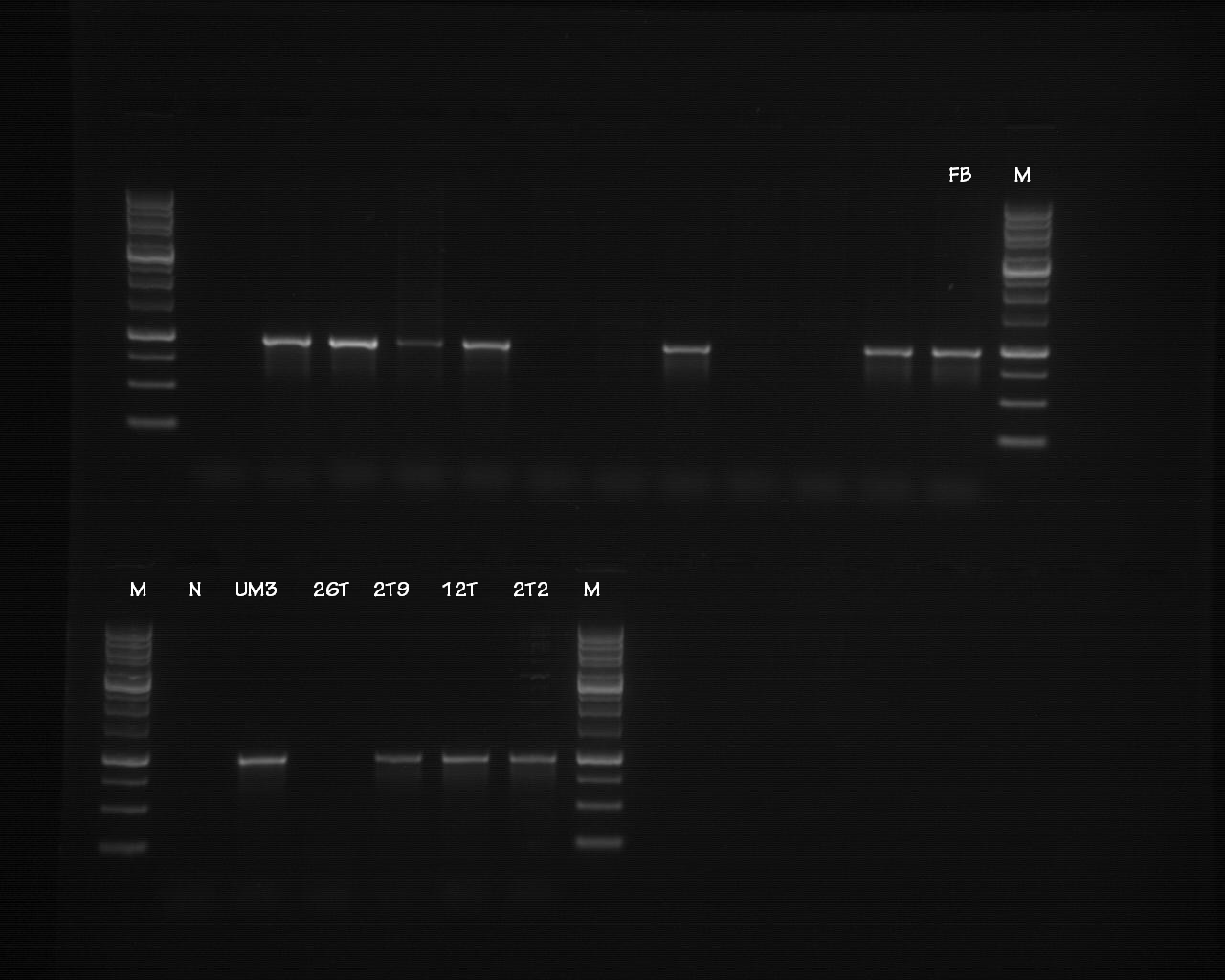

Supplement: Supplemental Information 6 [file peerj-11-16002-s006.zip › Figure 4/Target B/Target B_FB labelled.png]

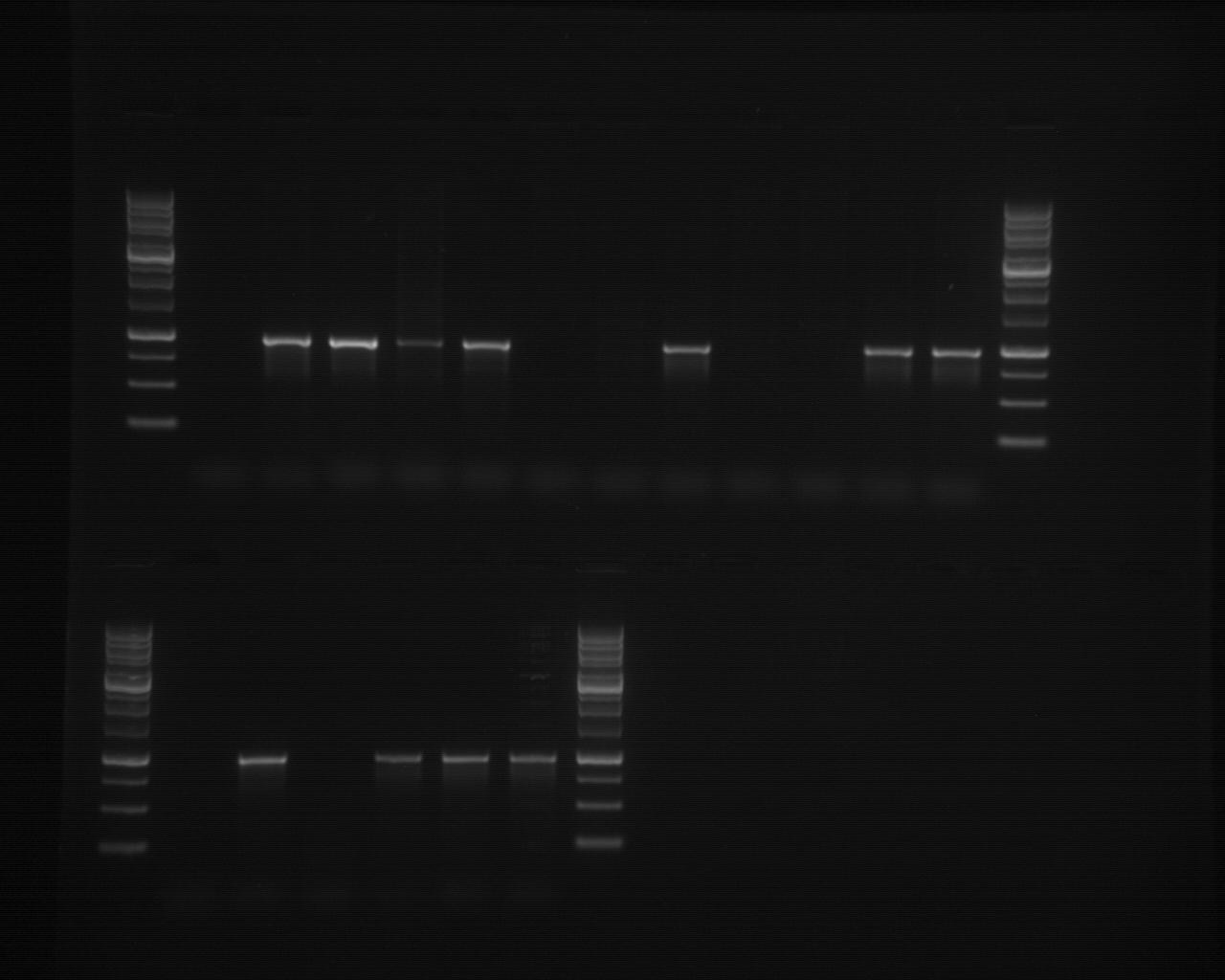

Supplement: Supplemental Information 6 [file peerj-11-16002-s006.zip › Figure 4/Target B/Target B_FB.png]

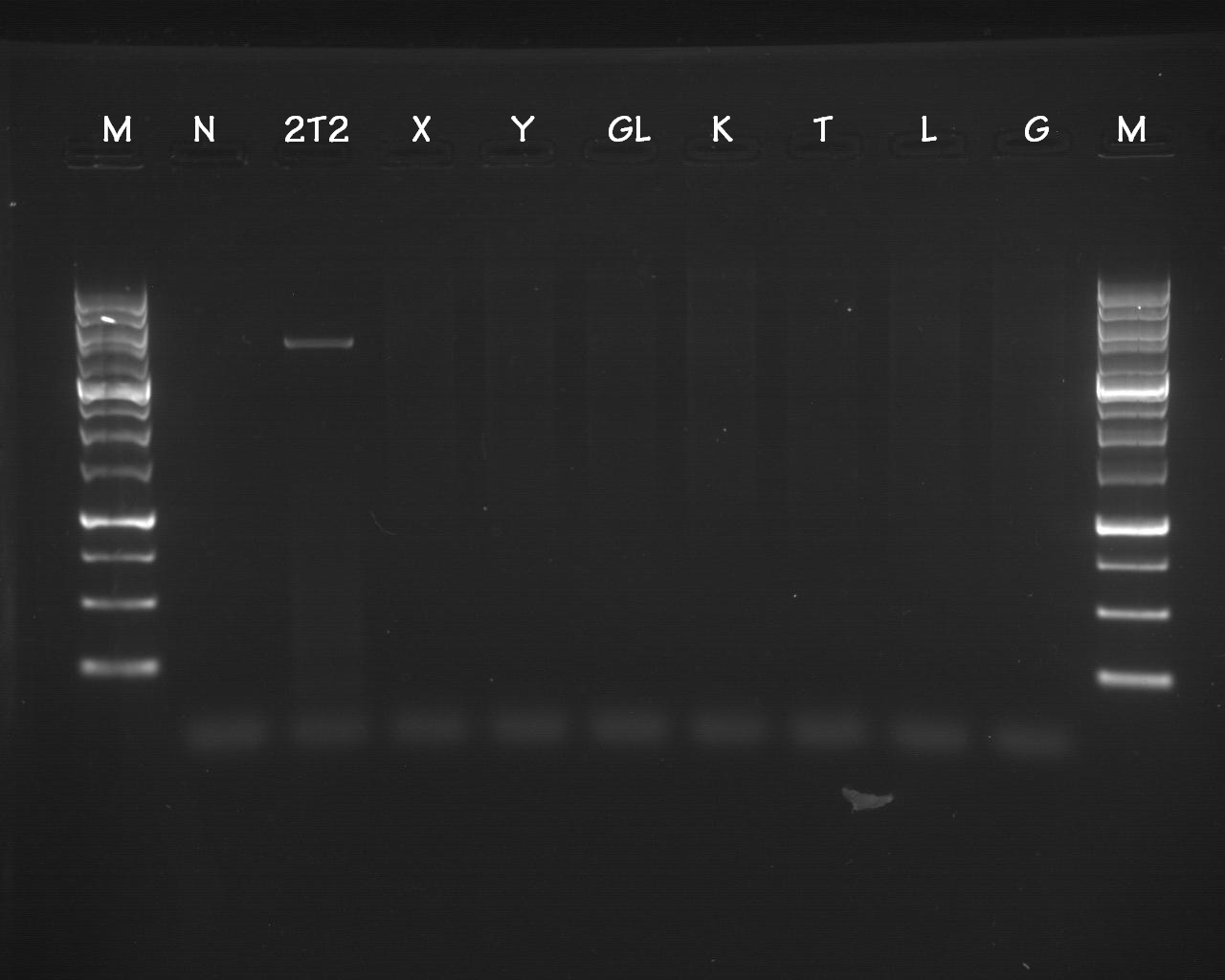

Supplement: Supplemental Information 6 [file peerj-11-16002-s006.zip › Figure 4/Target C/Target C labelled.png]

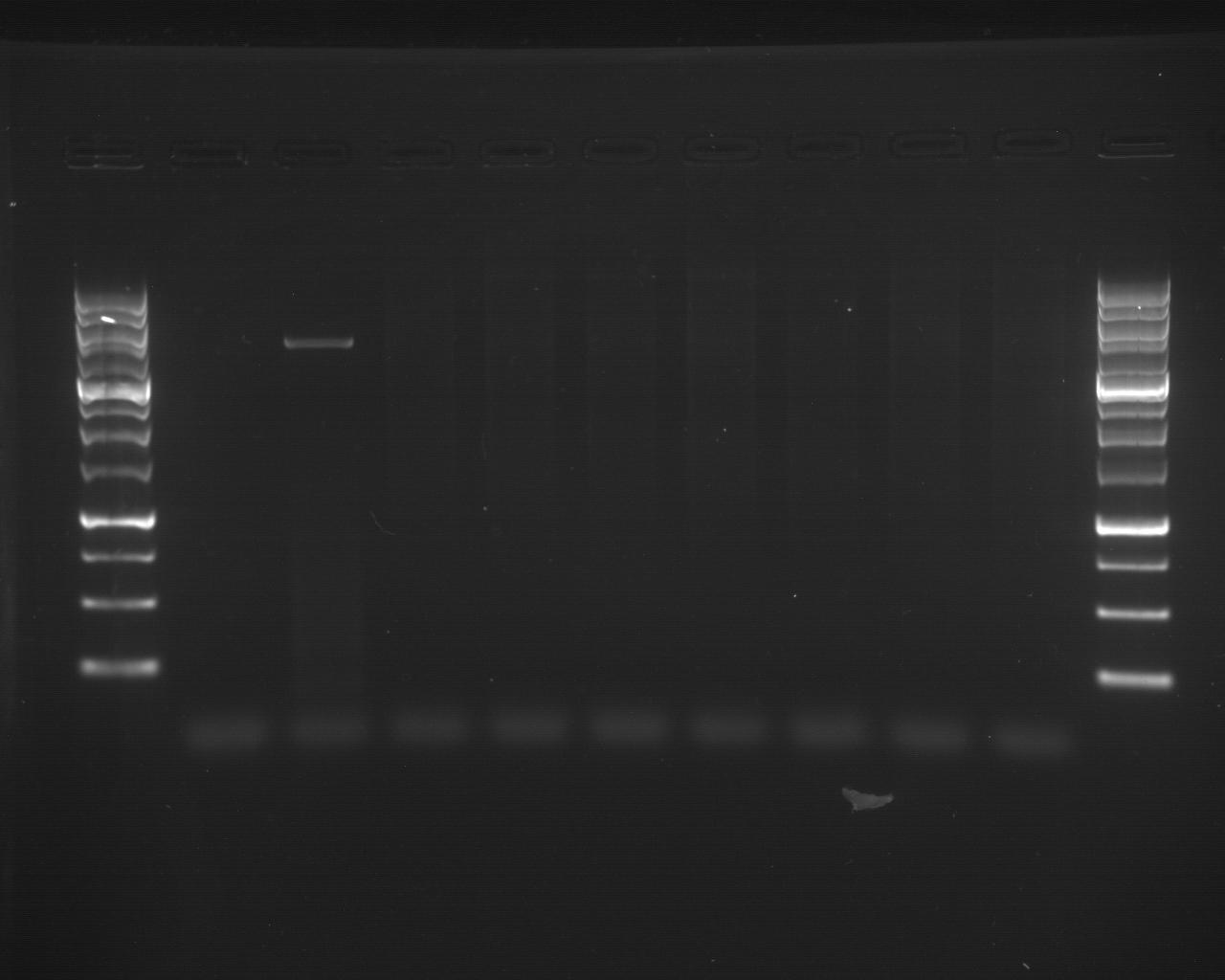

Supplement: Supplemental Information 6 [file peerj-11-16002-s006.zip › Figure 4/Target C/Target C.png]

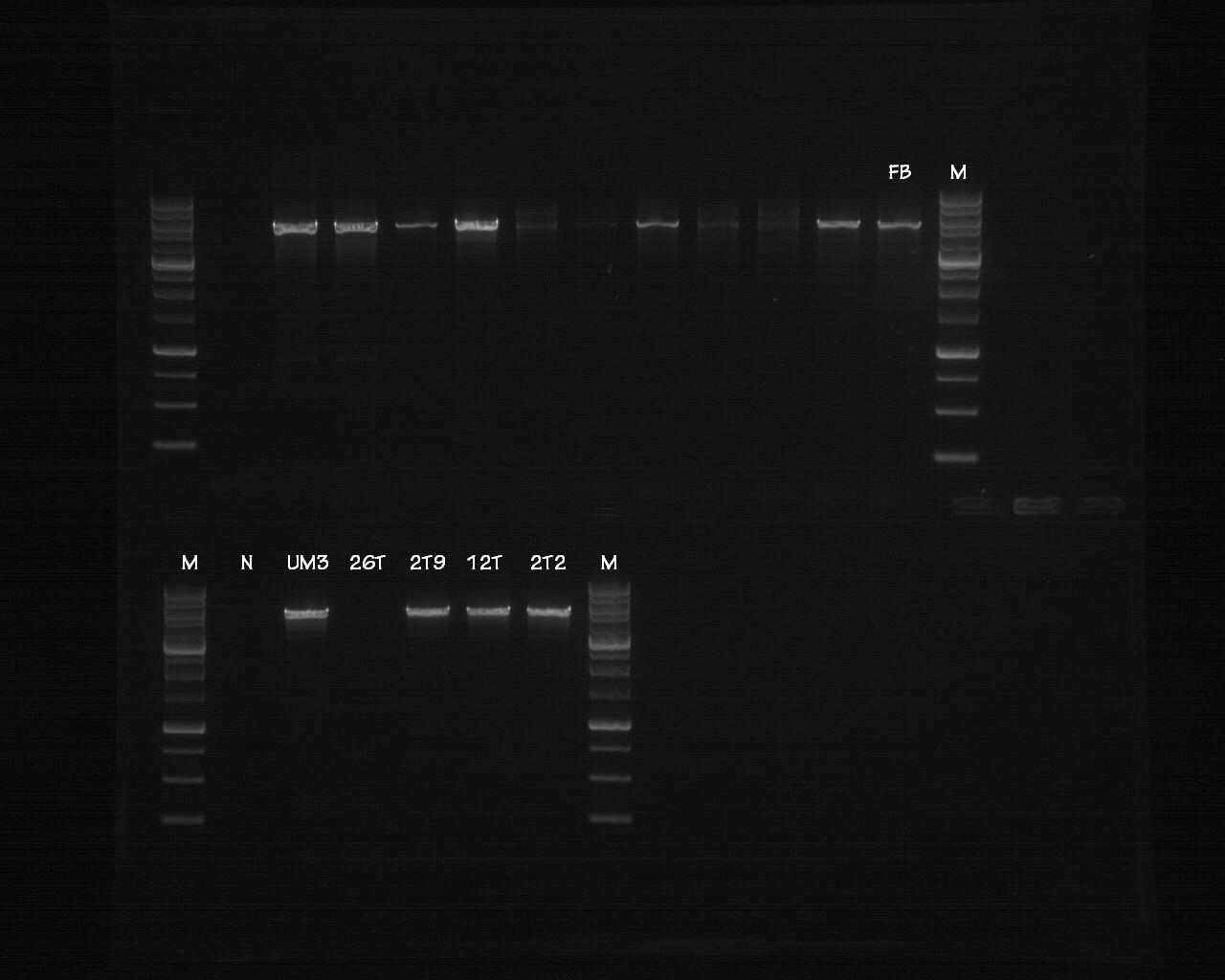

Supplement: Supplemental Information 6 [file peerj-11-16002-s006.zip › Figure 4/Target C/Target C_FB labelled.png]
